# Real-world safety profile of roflumilast: a pharmacovigilance analysis using FDA adverse event reporting system and Canada vigilance database

**DOI:** 10.3389/jpps.2025.15678

**Published:** 2026-01-12

**Authors:** Rui Xu, Hui Peng, Chang Shu, Maochang Liu, Ping Gao

**Affiliations:** Department of Pharmacy, Wuhan Children’s Hospital (Wuhan Maternal and Child Healthcare Hospital), Tongji Medical College, Huazhong University of Science and Technology, Wuhan, China

**Keywords:** roflumilast, adverse events, disproportionality analysis, FAERS, CVARD

## Abstract

**Background:**

Roflumilast, a highly selective phosphodiesterase 4 inhibitor, is used to treat with chronic obstructive pulmonary disease and psoriasis. We aim to determine potential roflumilast-associated adverse events (AEs) and the differences in AE signals among diverse populations.

**Methods:**

Roflumilast’s AE reports between the first quarter of 2011 and the fourth quarter of 2024 were obtained from the FDA Adverse Event Reporting System (FAERS) and Canada Vigilance Adverse Reaction Database (CVARD). The signal strength was measured by four disproportionality analysis methods, including Reporting Odds Ratio (ROR), Proportional Reporting Ratio (PRR), Bayesian Confidence Propagation Neural Network (BCPNN), and Multi-item Gamma Poisson Shrinker (MGPS).

**Results:**

In FAERS, population aged ≥65 years and oral medication users accounted for a predominant proportion in the reported cases. FDA-unlabeled respiratory, thoracic and mediastinal disorders was the only one signal categorized by system organ class met all four algorithms. Newly identified AEs such as dyspnea, condition aggravated, cough, and tachycardia could contribute valuable safety considerations for clinical practice. The analysis of the available time-to-onset data suggested that cases often occurred within the first 30 days post-treatment. These results were externally validated in CVARD, suggesting consistent findings. Notably, headache was more frequently reported among users of topical formulations and female patients, while suicidal ideation and weight loss were more commonly reported in male patients and oral medications, respectively.

**Conclusion:**

This study confirmed established adverse reactions and identified novel AEs in real-world clinical practice by dual-database pharmacovigilance analysis. Clinicians should remain vigilant for AEs that differ by gender and route to enable early intervention and improve prognosis. The findings highlight personalized safety management, while underscoring the necessity of prospective studies to validate results and further characterize roflumilast’s safety profile.

## Introduction

Roflumilast is the first phosphodiesterase 4 (PDE4) inhibitor indicated for chronic obstructive pulmonary disease (COPD) in 2011, subsequently for plaque psoriasis approved by the U.S. Food and Drug Administration (FDA) and Health Canada [1, 2]. Notably, COPD has been found to be a complication of psoriasis [3]. T lymphocytes play an important role in the immunopathogenesis of both psoriasis and COPD, driving chronic inflammation [4, 5]. These two diseases impact millions of individuals worldwide and represent a huge economic burden [6, 7]. PDE4 expression is higher in patients with inflammatory conditions than in healthy people [8]. The PDE4 enzyme family (isoforms PDE4A-D) specifically catalyzes the hydrolysis of cyclic adenosine monophosphate (cAMP) [9]. Roflumilast elevates cAMP levels in inflammatory and immune cells to reduce the release of inflammatory factors, such as tumor necrosis factor α (TNFα), and interleukin (IL)-17, thereby improving hyperactive immune responses [10].

Phase III clinical trials have demonstrated good efficacy and tolerability profile of roflumilast [11, 12]. However, safety concerns could limit roflumilast’s clinical use [13]. Immune-related disease treatment is a prolonged procedure. Continuous safety monitoring is essential to fully understand roflumilast’s adverse events (AEs) during treatment [14]. Nevertheless, large-scale data analysis of roflumilast-related adverse reactions in the real world is still insufficient. An analysis based on the U.S. MarketScan database identified potential safety signals through investigating roflumilast’s concomitant medications [15]. Our study draws on FDA and Canadian pharmacovigilance databases, facilitating the capture of a broader spectrum of global AE reports, meanwhile, providing a novel perspective on signal detection that differs from prescription sequence analysis.

The FDA Adverse Event Reporting System (FAERS) and Canada Vigilance Adverse Reaction Database (CVARD) are spontaneous reporting databases that monitor post-marketing drug safety [16, 17]. To provide a clinical rationalization of drug administration, we conducted disproportionality analyses to identify potential AEs associated with roflumilast and systematically evaluated adverse reactions across different patient subgroups.

## Methods

### Data source and processing

**FIGURE 1:**
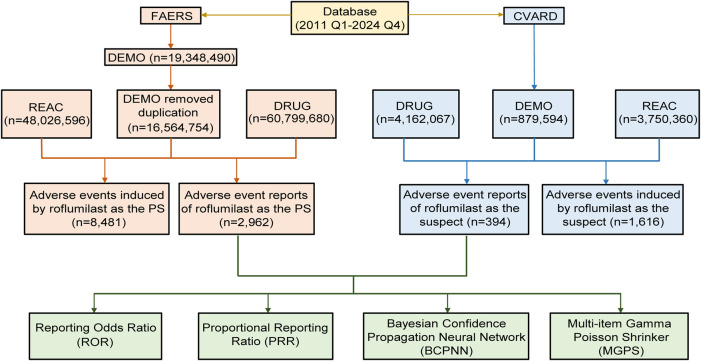
The flow diagram of screening roflumilast-related AE reports from FAERS and CVARD databases. FAERS, FDA Adverse Event Reporting System; CVARD, Canada Vigilance Adverse Reaction Database; DEMO, demographics; DRUG, drug information; REAC, adverse events; PS, primary suspect.

The FAERS and CVARD are publicly available pharmacovigilance databases for detecting new drug safety signals. They contain structured data fields, including demographics (DEMO), drug information (DRUG), adverse events (REAC), report sources (RPSR), and indication for use (INDI). Spontaneous AE reports are gathered from patients, healthcare providers, and others [16]. Using the medicine’s generic name (roflumilast) and trade name (DALIRESP, DAXAS, ZORYVE), we downloaded AE reports relevant to roflumilast from the first quarter (Q1) of 2011 to the fourth quarter (Q4) of 2024. Roflumilast was collected as the primary suspect (PS) from the FAERS and suspect in CVARD. The data were extracted, processed, and analyzed according to Figure 1. Following FDA guidelines, we performed data deduplication grounded in CASEID, FDA_DT, and PRIMARYID [18]. Then, we standardized AE terminology using Medical Dictionary of Regulatory Activities (MedDRA 27.1), which classifies events by System Organ Class (SOC) and Preferred Term (PT) levels. Possible indications (e.g., chronic obstructive pulmonary disease, bronchitis chronic, psoriasis) for roflumilast treatment as well as reactions (e.g., off-label use, intentional product misuse, medication error) unrelated to drug therapy were excluded before analyzing AEs.

A novelty/unexpected signal is defined as any positive adverse drug event detected that isn’t outlined in either the FDA or Canadian drug labels. The comparative methodology is as follows: First, AEs documented in the latest FDA drug labels and Canadian product monographs were systematically collated. Subsequently, Microsoft Excel 2021 was utilized to match these AEs with the preferred terms in the FAERS and CVARD. For AEs with different expressions but similar medical connotations, those with consistent or highly similar core meanings were identified as label-documented AEs following medical evaluation.

To evaluate the independent real-world safety profile of roflumilast, we conducted sensitivity analyses that excluded AE reports involving concomitant use with long-acting β_2_-agonists (e.g., formoterol), dual therapy (e.g., budesonide/formoterol, mometasone/formoterol, and fluticasone/formoterol), or triple therapy (e.g., budesonide/formoterol/glycopyrrolate and fluticasone/umeclidinium/vilanterol). This method could minimize potential effects from co-administered medications and enhance the specificity of AEs attribution to roflumilast therapy.

### Time-to-onset and weibull distribution analysis

We calculated the time-to-onset (TTO) of AEs as the interval between roflumilast initiation and AE occurrence. TTO data were summarized using median with interquartile range (IQR). We employed Weibull distribution modeling (parameters α and β) to characterize time-dependent risk patterns [19]. The scale parameter (α) indicates the spread of distribution, whereas the shape parameter (β) describes the curve’s form. An early failure-type pattern is indicated when β < 1 and its 95% confidence interval (CI) < 1, suggesting a decreasing risk over time. A random failure-type pattern is inferred when β is close to 1 and its 95% CI includes 1, reflecting a constant hazard. A wear-out failure-type pattern is identified when β > 1 and its 95% CI excludes 1, indicating an increasing risk over the course of treatment.

### Statistical analysis

In this study, we utilized four standard disproportionality metrics: Reporting Odds Ratio (ROR), Proportional Reporting Ratio (PRR), Bayesian Confidence Propagation Neural Network (BCPNN), and Multi-item Gamma Poisson Shrinker (MGPS). The two-by-two contingency table formulas and detailed calculation methods are provided in Supplementary Tables S1, S2. Significant signals that met at least one of the four analysis methods were considered as roflumilast-associated positive AEs. Among the four methods, the ROR performs well in sensitivity and early detection, the PRR offers high specificity, the BCPNN supports multi-source data integration and cross-validation, and the MGPS excels in identifying signals for rare events [20, 21]. This study comprehensively applies the four algorithms to detect drug safety signals more comprehensively and reliably. Higher values suggested a stronger link between the drug and the occurrence of AEs. Due to the small number of reports with roflumilast in CVARD database, subgroup analysis results couldn’t be effectively output. Subgroup analyses were performed for reports in the FAERS database (Supplementary Table S3). A lower limit of the 95% CI of the adjusted ROR >1 indicated greater probability of AE relevance in the target group, while 0 < 95% CI of the adjusted ROR <1 suggested higher relevance in the control group. The p value was corrected by the false discovery rate method. Adjusted p value (p.adj) < 0.05 indicated that the difference was statistically significant. R software (version 4.4.2) was applied for all statistical assessments.

## Results

### Descriptive characteristics

From 2011 Q1 to 2024 Q4, 19,348,490 cases were obtained from the FAERS database. After dereplication, this study ultimately contained 2,962 AE reports related to roflumilast and 8,481 roflumilast-associated AEs (Figure 1). Clinical features of AE reports related to roflumilast are described in the Table 1. Males made up 45.7% (n = 1,354) of these reports, while females accounted for 41.2% (n = 1,221). 51.6% of the age information reports was absent. 13.3% (n = 395) of all reports were from those aged 18 to 64, and 34.9% (n = 1,034) were over 65 years. Oral administration accounted for 89.5% (n = 2,650) of total reports, while topical usage was 7.7% (n = 228). Unfortunately, weight information was missing in 73% of the reports. The percentage of people weighing between 50 and 100 kg was comparatively higher, making up 21.4%. Healthcare professionals and consumers were the main reporters. COPD and psoriasis were the main indications. The top five countries with the most reports were the United States, Germany, Canada, South Korea, and Netherlands. According to reporting years, 2013 had the highest percentage of reports (26.4%), followed by 2014 (10.3%), 2023 (9.7%), 2012 (7.3%), and 2024 (7.2%).

**TABLE 1:** Characteristics of roflumilast-related adverse event reports in the FAERS database (2011 Q1 to 2024 Q4).

Characteristics	Case numbers	Case proportion
Number of reports	2,962	​
Gender		
Male	1,354	45.7%
Female	1,221	41.2%
Missing	387	13.1%
Age		
<18	6	0.2%
18–64	395	13.3%
≥65	1,034	34.9%
Missing	1,527	51.6%
Route		
Oral	2,650	89.5%
Topical	228	7.7%
Other	84	2.8%
Weight		
<50 kg	90	3.0%
>100 kg	77	2.6%
50–100 kg	633	21.4%
Missing	2,162	73.0%
Reporters		
Healthcare professional	896	30.2%
Consumer	1,062	35.9%
Other	686	23.2%
Missing	318	10.7%
Indications (top 3)		
COPD	1,390	46.9%
Missing	1,279	43.1%
Psoriasis	86	2.9%
Reported countries (top 5)		
United States	2,275	76.8%
Germany	450	15.2%
Canada	32	1.1%
South Korea	25	0.8%
Netherlands	16	0.5%
Reporting year (top 5)		
2013	783	26.4%
2014	305	10.3%
2023	288	9.7%
2012	217	7.3%
2024	214	7.2%

### Signals detection at the SOC level

Following statistical analysis, we discovered that roflumilast-induced AEs occurrence targeted 26 organ systems. The top five SOCs by frequency were general disorders and administration site conditions (n = 1,481), gastrointestinal disorders (n = 1,257), psychiatric disorders (n = 983), respiratory, thoracic and mediastinal disorders (n = 898), and nervous system disorders (n = 736). Positive SOCs were included gastrointestinal disorders (ROR 1.85, PRR 1.72, EBGM 1.72, IC 0.78), psychiatric disorders (ROR 2.16, PRR 2.03, EBGM 2.03, IC 1.02), respiratory, thoracic and mediastinal disorders (ROR 2.34, PRR 2.20, EBGM 2.20, IC 1.14), investigations (ROR 1.14, PRR 1.13, EBGM 1.13, IC 0.18) and metabolism and nutrition disorders (ROR 1.66, PRR 1.63, EBGM 1.63, IC 0.71). Notably, only respiratory, thoracic and mediastinal disorders met all four criteria simultaneously (Table 2).

**TABLE 2:** Signal strength of adverse events of roflumilast at the SOC level.

SOC	Case number	ROR (95% Cl)	PRR (χ^2^)	EBGM (EBGM05)	IC (IC025)
General disorders and administration site conditions	1,481	0.99 (0.94, 1.05)	0.99 (0.05)	0.99 (0.95)	−0.01 (-0.09)
Gastrointestinal disorders^*^	1,257	1.85 (1.74, 1.96)^*^	1.72 (415.8)	1.72 (1.64)	0.78 (0.7)^*^
Psychiatric disorders^*^	983	2.16 (2.02, 2.31)^*^	2.03 (541.95)^*^	2.03 (1.92)	1.02 (0.92)^*^
Respiratory, thoracic and mediastinal disorders^*^	898	2.34 (2.19, 2.51)^*^	2.2 (617.68)^*^	2.2 (2.08)^*^	1.14 (1.04)^*^
Nervous system disorders	736	1.02 (0.95, 1.1)	1.02 (0.26)	1.02 (0.96)	0.03 (-0.09)
Investigations^*^	600	1.14 (1.05, 1.24)^*^	1.13 (10.17)	1.13 (1.06)	0.18 (0.06)^*^
Injury, poisoning and procedural complications	460	0.55 (0.5, 0.6)	0.57 (164.7)	0.57 (0.53)	−0.81 (-0.95)
Musculoskeletal and connective tissue disorders	378	0.83 (0.75, 0.92)	0.84 (12.48)	0.84 (0.77)	−0.25 (-0.41)
Infections and infestations	309	0.67 (0.6, 0.75)	0.68 (47.6)	0.68 (0.62)	−0.55 (-0.71)
Metabolism and nutrition disorders^*^	300	1.66 (1.48, 1.86)^*^	1.63 (75.59)	1.63 (1.48)	0.71 (0.54)^*^
Skin and subcutaneous tissue disorders	262	0.55 (0.49, 0.63)	0.57 (90.67)	0.57 (0.51)	−0.81 (-1)
Cardiac disorders	248	1.1 (0.97, 1.25)	1.1 (2.28)	1.1 (0.99)	0.14 (-0.05)
Vascular disorders	115	0.62 (0.52, 0.74)	0.62 (26.59)	0.62 (0.54)	−0.68 (-0.95)
Neoplasms benign, malignant and unspecified (incl cysts and polyps)	101	0.44 (0.36, 0.54)	0.45 (70.61)	0.45 (0.38)	−1.16 (-1.45)
Eye disorders	70	0.4 (0.32, 0.51)	0.41 (61.44)	0.41 (0.33)	−1.29 (-1.64)
Renal and urinary disorders	56	0.35 (0.27, 0.46)	0.36 (66.28)	0.36 (0.29)	−1.49 (-1.87)
Social circumstances	34	0.91 (0.65, 1.28)	0.91 (0.27)	0.91 (0.69)	−0.13 (-0.62)
Immune system disorders	32	0.33 (0.24, 0.47)	0.34 (42.45)	0.34 (0.25)	−1.57 (-2.08)
Blood and lymphatic system disorders	31	0.21 (0.15, 0.3)	0.21 (92.08)	0.21 (0.16)	−2.23 (-2.75)
Reproductive system and breast disorders	27	0.38 (0.26, 0.56)	0.39 (26.62)	0.39 (0.28)	−1.37 (-1.92)
Surgical and medical procedures	27	0.23 (0.16, 0.34)	0.23 (69.45)	0.23 (0.17)	−2.11 (-2.65)
Hepatobiliary disorders	26	0.33 (0.22, 0.49)	0.33 (35.16)	0.33 (0.24)	−1.59 (-2.14)
Ear and labyrinth disorders	24	0.65 (0.44, 0.97)	0.65 (4.46)	0.65 (0.47)	−0.62 (-1.19)
Product issues	19	0.14 (0.09, 0.22)	0.14 (101.5)	0.14 (0.1)	−2.83 (-3.48)
Endocrine disorders	6	0.27 (0.12, 0.61)	0.28 (11.49)	0.28 (0.14)	−1.86 (-2.95)
Congenital, familial and genetic disorders	1	0.04 (0.01, 0.27)	0.04 (24.15)	0.04 (0.01)	−4.7 (-6.74)

SOC, system organ class; ROR, reporting odds ratio; PRR, proportional reporting ratio; EBGM, empirical bayesian geometric mean; EBGM05, the lower limit of the 95% CI of EBGM; IC, information component; IC025, the lower limit of the 95% CI of the IC; CI, confidence interval.

Asterisks (*) indicate positive signals.

### Risk signals analyses at the PT level

The signals for the top 100 PTs ranked by frequency were presented in Supplementary Table S4. We found some side effects that were mentioned in the instructions but rarely reported, such as rash (n = 31), influenza (n = 16), nasopharyngitis (n = 14), and neoplasm malignant (n = 12).

**FIGURE 2:**
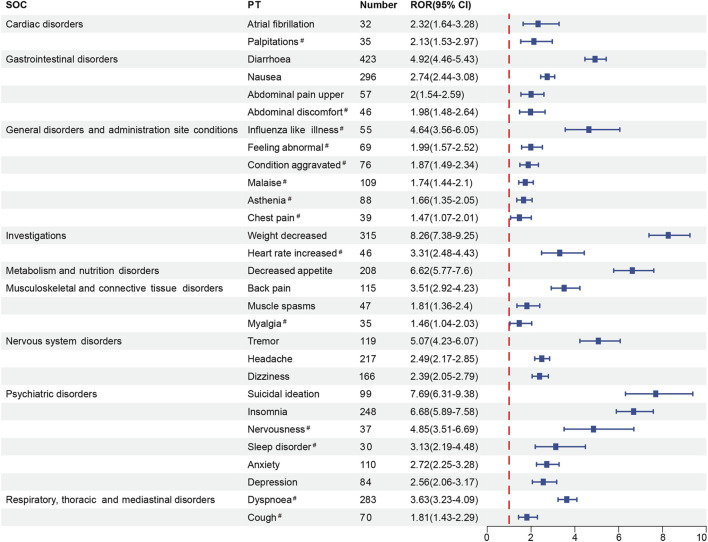
The forest plot of positive signals (n ≥ 30) related to roflumilast at the PT level. SOC, System Organ Class; PT, Preferred Term; ROR, Reporting Odds Ratio; CI, confidence interval. Hashtags (^#^) indicate AEs not marked in the FDA-drug’s label.

Among positive PTs with ≥30 reported cases, half of these PTs were marked in the drug’s label, containing diarrhea (n = 423, ROR 4.92), weight decreased (n = 315, ROR 8.26), nausea (n = 296, ROR 2.74), insomnia (n = 248, ROR 6.68), headache (n = 217, ROR 2.49), decreased appetite (n = 208, ROR 6.62), dizziness (n = 166, ROR 2.39), tremor (n = 119, ROR 5.07), back pain (n = 115, ROR 3.51), anxiety (n = 110, ROR 2.72), suicidal ideation (n = 99, ROR 7.69), depression (n = 84, ROR 2.56), upper abdominal pain (n = 57, ROR 2.00), muscle spasms (n = 47, ROR 1.81) and atrial fibrillation (AF, n = 32, ROR 2.32). In addition, 14 potential adverse reactions were FDA-unlabeled, including dyspnea (n = 283, ROR 3.63), malaise (n = 109, ROR 1.74), asthenia (n = 88, ROR 1.66), condition aggravated (n = 76, ROR 1.87), cough (n = 70, ROR 1.81), feeling abnormal (n = 69, ROR 1.99), influenza-like illness (n = 55, ROR 4.64), heart rate increased (n = 46, ROR 3.31), abdominal discomfort (n = 46, ROR 1.98), chest pain (n = 39, ROR 1.47), nervousness (n = 37, ROR 4.85), palpitations (n = 35, ROR 2.13), myalgia (n = 35, ROR 1.46), and sleep disorder (n = 30, ROR 3.13) (Figure 2).

### Stratified subgroup analysis

**FIGURE 3:**
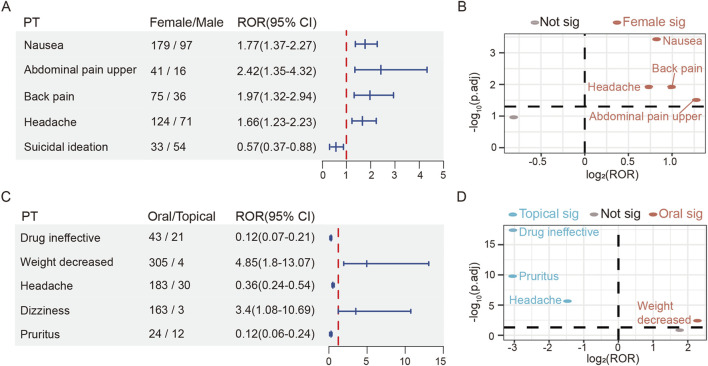
The risk differences of roflumilast in gender and route subgroup. **(A)** The forest plot of adjusted ROR for gender-related AEs. **(B)** Gender-differentiated risk signals volcano plot for roflumilast. **(C)** The forest plot of adjusted ROR for route-related AEs. **(D)** Route-differentiated risk signals volcano plot for roflumilast. SOC, System Organ Class; PT, Preferred Term; ROR, Reporting Odds Ratio; CI, confidence interval.

Gender-stratified analyses were performed on the 50 most frequent AEs (Supplementary Tables S5, S6). Of the top 50 signals, pruritus (n = 19), depressed mood (n = 12), and influenza (n = 11) reported only in the female population, while AF (n = 22) and suicide attempt (n = 13) required extra clinical surveillance in male patients. Our analysis suggested a potential association between males and suicidal ideation (ROR 0.57, 95% CI: 0.37–0.88), but no significant sex-based differentiation was observed. Notably, comparative analyses revealed that females exhibited significantly higher risk of headache (ROR 1.66, 95% CI: 1.23–2.23), nausea (ROR 1.77, 95% CI: 1.37–2.27), back pain (ROR 1.97, 95% CI: 1.32–2.94), and upper abdominal pain (ROR 2.42, 95% CI: 1.35–4.32) than male patients (Figures 3A,B).

Among the top 50 PTs according to age subgroup analyses, patients over 64 had higher risk of decreased appetite rather than those within the group of 18–64 years (ROR 1.61, 95% CI: 1.08–2.39). The incidences of headache (ROR 0.67, 95% CI: 0.47–0.95) and suicidal ideation (ROR 0.52, 95% CI: 0.31–0.89) should be monitored in the group aged 18–64. Although these reactions suggest different associations of adverse effects by age group, there is no effective difference (Supplementary Figure S1). The <18 age group contained insufficient PTs for robust statistical analysis, it might need attention to respiratory distress, sensitive skin, and gastritis. Signal values for age-related analyses are provided in Supplementary Tables S7–S9.

The top 50 prominent AEs were also subjected to route subgroup analyses. Both oral and topical therapy with roflumilast could cause diarrhea, nausea, insomnia, headache, heart rate increased, rash, and pruritus. Additionally, it was revealed that orally treated patients required additional attention, including decreased appetite (n = 204), anxiety (n = 107), suicidal ideation (n = 97), AF (n = 32) and sleep disorder (n = 29). On the other hand, topical group requested caution for skin burning sensation (n = 15), skin exfoliation (n = 10), application site pain (n = 8), dermatitis contact (n = 7), urinary tract infection (n = 5), skin discoloration (n = 4), and urticaria (n = 4) (Supplementary Tables S10, S11). Compared to the topically treated group, the oral group showed a higher adjusted ROR signal strength for weight decreased (ROR 4.85, 95% CI: 1.8–13.07). However, topical preparation was more likely to cause headache (ROR 0.36, 95% CI: 0.24–0.54), pruritus (ROR 0.12, 95% CI: 0.06–0.24) and drug ineffective (ROR 0.12, 95% CI: 0.07–0.21) (Figures 3C,D).

### TTO and weibull distribution analysis

**FIGURE 4:**
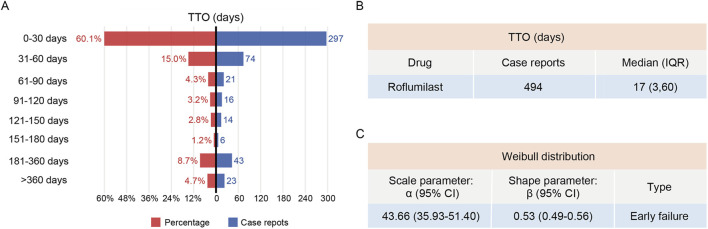
TTO and Weibull distribution analysis of roflumilast-induced adverse reactions. **(A)** The frequency and percentage distribution of TTO reports across various time periods. **(B)** TTO of roflumilast-induced adverse reactions. **(C)** Weibull distribution analysis of TTO reports. TTO, time-to-onset; IQR, interquartile range; CI, confidence interval.

The data on AE onset were available for only 494 reports (16.7%) in the FAERS database. The analysis of the available reports with complete TTO data suggested that cases often occurred within the first 30 days after roflumilast administration, and then the incidence of AEs decreased gradually (Figure 4A). Moreover, TTO data suggested a median onset time of 17 days and an IQR of 3–60 days (Figure 4B). Weibull distribution analysis of roflumilast-associated AEs revealed an early failure pattern, indicating decreasing AEs incidence over time (Figure 4C).

### Sensitivity analysis

Roflumilast is frequently prescribed as add-on therapy to long-acting bronchodilator, including formoterol, dual therapy (e.g., budesonide/formoterol, mometasone/formoterol, and fluticasone/formoterol), or triple therapy (e.g., budesonide/formoterol/glycopyrrolate and fluticasone/umeclidinium/vilanterol). After excluding these commonly co-administered drugs, a reanalysis of the top 100 PT signals was performed in Supplementary Table S12. The identified adverse reactions almost corresponded to previous findings, such as psychiatric symptoms, gastrointestinal diseases, respiratory tract disorders, and skin problems.

### External validation in CVARD

**FIGURE 5:**
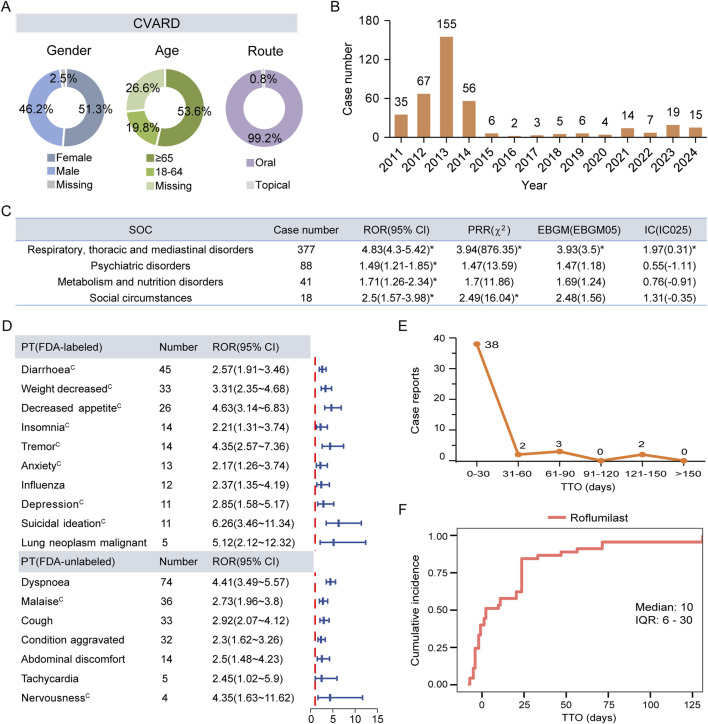
External validation using the CVARD. **(A)** Basic features of AE reports for roflumilast. **(B)** Temporal distribution of adverse drug event reports from 2011 Q1 to 2024 Q4. **(C)** Positive signals detection at the SOC level. **(D)** The forest plot of significant ROR signals at the PT level. **(E)** The frequency distribution of TTO reports. **(F)** The Kaplan-Meier curve depicts the cumulative incidence. CVARD, Canada Vigilance Adverse Reaction Database; SOC, System Organ Class; ROR, Reporting Odds Ratio; PRR, Proportional Reporting Ratio; EBGM, Empirical Bayesian Geometric Mean; EBGM05, the lower limit of the 95% CI of EBGM; IC, Information Component; IC025, the lower limit of the 95% CI of the IC; CI, confidence interval. PT, Preferred Term; TTO, time-to-onset; IQR, interquartile range. Asterisks (^*^) indicate positive signals. Alphabets(^C^) indicate AEs documented in the Canadian Product Monographs.

A total of 394 reports were linked to roflumilast-related AEs in the CVARD database from 2011 to 2024 (Figure 1). Our analysis showed a comparable proportion of male (46.2%) and female (51.3%) patients treated with roflumilast, with the highest number of cases occurring in individuals aged ≥65 years (53.6%) or receiving oral formulation (99.2%) (Figure 5A). In 2013, the total number of reports (n = 155) exceeded those of any other year (Figure 5B). At the SOC level, respiratory, thoracic and mediastinal disorders (n = 377, ROR 4.83, PRR 3.94, EBGM 3.93, IC 1.97) was the only positive signal that matched the four algorithms (Figure 5C). These findings were consistent with FAERS data.

Further analysis of ROR-positive AEs revealed that 15 PTs overlapped between the Top 30 signals in FAERS and the Top 40 signals in CVARD. The identified AEs comprised 8 labeled events and 7 FDA-unlabeled potential safety concerns. These potential AEs included dyspnea (n = 74, ROR 4.41), malaise (n = 36, ROR 2.73), cough (n = 33, ROR 2.92), condition aggravated (n = 32, ROR 2.3), abdominal discomfort (n = 14, ROR 2.5), tachycardia (n = 5, ROR 2.45), and nervousness (n = 4, ROR 4.35). In contrast to the FDA-approved labeling, the Canadian product monograph lists malaise and nervousness as documented AEs. Influenza (n = 12, ROR 2.37) and lung neoplasm malignant (n = 5, ROR 5.12) were identified among the top 40 significant PTs in CVARD. However, these events are documented in the FDA-approved labeling but absent from the Canadian product monograph (Figure 5D; Supplementary Figure S2). Consistent with FAERS data, AE reporting frequency of CVARD peaked within the first 30 days post-administration period followed by a gradual decline (Figure 5E). The median TTO for roflumilast-mediated adverse reactions was 10 days (IQR: 6–30 days) (Figure 5F).

## Discussion

In this study, we systematically analyzed the real-world reports of roflumilast from 2011 Q1 to 2024 Q4 through the FAERS and CVARD databases. Analysis of patient characteristics revealed that the AE reporting rates were higher among the older adults (≥65 years) and oral administration population. The data suggested that similar counts of roflumilast’s adverse reaction reports were observed in males and females. Roflumilast-related AEs were reported most frequently in 2013, showing the first peak. This may be attributed to the Weber effect, which describes the characteristic pattern of spontaneously reported adverse reactions peaking within the first 2 years post-approval and subsequently declining [22]. While the second peak was in 2023, following its application for psoriasis. These results highlight the extensive clinical application of roflumilast and the requirement for surveillance.

We found some adverse reactions contained in the drug instructions, including diarrhea, nausea, decreased appetite, weight loss, headache, dizziness, insomnia, anxiety, depression, suicidal ideation and lung neoplasm malignant, which validated the reliability of our results. Our analysis identified distortional reporting of roflumilast-associated AEs that were not documented in the prescribing information, such as heart rate increased, tachycardia, palpitations, and condition aggravated.

The meta-analysis showed that AF was more frequent in the roflumilast group than in the placebo group (0.4% vs. 0.2%) [23]. An accelerated and irregular heart rhythm is the hallmark of AF, which can be non-symptomatic or cause symptoms like palpitations, increased heart rate, chest pain, nausea, dizziness, dyspnea, and general fatigue [24]. Immune remodeling is an important mechanism in AF which can increase the release of cytokines (e.g., IL-6) and exosomes by activating macrophages. In turn, pacing cardiomyocytes may further promote macrophage activation [25]. PDE4, a key factor in the regulation of heart rate, is able to alter cAMP concentrations of cardiac ryanodine receptor 2 microstructural domain. Likewise, the upregulation of PDE4-dependent cAMP levels in sinus node myocytes increases heart rate [26, 27]. It has been shown that administration of the high-dose PDE4 inhibitor rolipram significantly increased heart rate [28]. In chronic kidney disease, elevated levels of fibroblast growth factor 23 inhibited PDE4B expression in cardiomyocytes, further increasing sarcoplasmic reticulum Ca^2+^ leakage as well as promoting ventricular arrhythmias [29]. Roflumilast exacerbated tachycardia and hypotension in septic rats while improving renal perfusion and liver damage [30]. Notably, knockdown of PDE4D increased heart rate in hypertensive mice, helping them respond to β-adrenergic receptor stimulation of the sinus node normally [26]. The mechanisms by which roflumilast induces cardiac AEs like AF and palpitations are not yet fully understood, whereas animal studies suggest several potential pathways mediated by PDE4 inhibition. COPD or psoriasis patients who are experiencing hypertension may benefit from roflumilast medication. For patients with chronic nephritis and sepsis, blood pressure and electrocardiogram should be monitored regularly after applying roflumilast.

AEs that strongly associated with roflumilast were condition aggravated, chest pain, influenza-like illness, dyspnea, and cough, despite these actions being not labeled in the insert. Few studies have found the existence of the above five adverse actions. The DERMIS-2 trial observed that one case of psoriasis progression was reported in the topical roflumilast group [12]. In a randomized controlled trial utilizing oral roflumilast for the treatment of moderate-to-severe psoriasis, a non-ischemic chest pain event occurred at weeks 12–24 [31]. A pooled analysis showed that topical roflumilast caused upper respiratory tract infections [32]. CHEST guideline suggests that respiratory tract infections are a major cause of cough [33]. In a 16-week, randomized, double-blind, placebo-controlled trial, a lower number of serious AEs experienced in the roflumilast treatment group (n = 8), including COPD worsening, dyspnea, and influenza A virus. Meanwhile, cough belonged to mild-to-moderate side effect [34]. Although the exact mechanism of exacerbations during roflumilast treatment remains unknown, we hypothesize that it might be connected to the dual effects of cAMP, which reduces T cell proliferation by facilitating the release of IL-10. However, higher dosages of roflumilast caused bone marrow-derived dendritic cells to elevate PDE4B and PDE4D expressions in response to increased cAMP levels. The result was followed by Th17 cell polarization and high expression of IL-23 that stimulated the release of IL-17 and IL-6 [35]. It is crucial to monitor the changes of inflammatory cytokine levels before and after the clinical application of roflumilast, considering the prognosis and quality of life of patients. Physicians should timely detect and assess exacerbation, chest pain, and respiratory dysfunctions in roflumilast-treated patients. Depending on the situation, adjusting the therapeutic dosage, the combination of medications, or symptomatic supportive therapy may be required.

Multiple clinical trials demonstrated that the roflumilast-related predominant AEs involved the gastrointestinal and psycho-neurological systems, as well the majority of AEs were transient and mild-to-moderate in severity. Long-term treatment of roflumilast in COPD or plaque psoriasis revealed higher incidence rates of diarrhea, nausea, headache, and insomnia than placebo [1, 36]. An analysis of 15 trials involving 11,168 COPD subjects found higher incidences of psychiatric-related adverse reactions in roflumilast 500 μg group than those in the control group (OR 2.13, 95% CI: 1.79–2.54), particularly an increased possibility of sleep disorder, anxiety, and depressed mood. Notably, participants receiving roflumilast reported 3 cases of suicidal behavior and 2 suicide attempts, whereas no related behaviors were noted in placebo-treated participants. No statistically significant difference was observed in psychiatric AEs between the 250 μg roflumilast and placebo groups (OR 0.87, 95% CI: 0.56–1.33) [37]. Although nervousness and sleep disorders are not listed in the FDA-approved labeling while being included in Canadian labels, their identification in our real-world data illustrates the dynamic nature of drug safety knowledge, which in turn underscores the importance of ongoing, systematic pharmacovigilance to capture and validate safety signals across diverse information sources.

Present studies have found that the regulatory role of PDE4 in gastrointestinal systems strongly associated with common AEs, such as nausea and diarrhea. The following postulated mechanisms may contribute to these two AEs. There is evidence that nausea is closely connected to delayed stomach transit. Roflumilast inhibited gastric transit more effectively than selective PDE4B inhibitors, but PDE4 inhibitor-induced gastroparesis wasn’t influenced by gene deletion of any one PDE4 subtype, illustrating that two or more PDE4 subtypes may be involved in this condition. Mechanistically, roflumilast-induced PDE4D inhibition may contribute to diarrhea through the signals of cystic fibrosis transmembrane conductance regulator and 5-hydroxytryptamine 4 receptor [38]. Animal research revealed that a prolonged overdose of roflumilast led to diarrhea, as well as increasing serum levels of cytokine-induced neutrophil chemotactic factor 1 and leucocytes. Diclofenac, a non-selective cyclooxygenase inhibitor, could protect against the toxic effects mentioned above by roflumilast [39]. Therefore, taking diclofenac helps patients improve roflumilast-induced diarrhea and abdominal pain. Anti-diarrheal drugs like oral rehydration salts can be used to treat dehydration induced by severe diarrhea. In addition, roflumilast inhaler is being developed to avoid gastrointestinal problems [40].

PDE4 also plays a critical role in the central nervous system. When compared to wild-type (WT) mice, PDE4A KO or PDE4B KO mice proved anxiety characteristics along with higher corticosterone levels, while PDE4B KO mice also showed increased long-term depression [41–43]. Furthermore, PDE4D KO mice slept for a shorter time than their WT littermates under xylazine/ketamine-induced anesthesia [44]. The above mechanistic hypothesis could be the reason for induction of insomnia, sleep disorder or vomiting with roflumilast. Thus, physicians should proactively inquire about the patient’s psychological history or conduct a psychological examination before prescribing roflumilast. To lower the patient’s risk of adverse mental events, dose reduction or gradual dose escalation should be considered.

In our study, there were only a limited number of reports regarding AEs associated with cancer. Reports of cancer in clinical trials were consistently rare and not considered to be directly caused by roflumilast [1]. Recent research has indicated that roflumilast may inhibit the proliferation of liver and ovarian cancer cells [45, 46]. However, PDE4-dependent cAMP was also found to suppress both innate and adaptive immunity, which could potentially facilitate immunological escape. Evidence suggested that roflumilast increased the volume of B-cell lymphomas while decreasing CD3^+^ cell and CD4/CD8 ratios, indicating its potential immunosuppressive properties. Specifically, inhibition of PDE4 enhanced cytokines (IL-10, IL-8, and IL-6) gene transcription through activation of the cAMP/PKA/CREB signaling pathway. These cytokines subsequently bond to their respective receptors in an autocrine manner, further phosphorylating JAK/STAT signaling and elevating transcription as well as surface expression levels of the immune checkpoint PD-L1 [47]. The aforementioned speculative mechanisms for roflumilast-associated malignancies require validation with additional clinical samples. Despite the ongoing debate on roflumilast’s carcinogenic potential, close surveillance of cancer-related AEs in clinical practice is indispensable.

Roflumilast plasma concentrations were similar among children, adolescents, and adults; however, gender and age were found to influence roflumilast clearance and metabolic fraction *in vivo*. A pharmacokinetic study of roflumilast revealed higher total PDE4 inhibitory activity in female and older patients with COPD [48, 49]. Multivariate analysis of Korean patients revealed that older age was significantly associated with higher rates of roflumilast treatment withdrawal [50]. Thus, it was considered that drug discontinuation is more likely to occur in females and the older adults. In terms of gender, females were prone to develop AEs such as nausea, headache, and back pain. Notably, males were more inclined to AF and suicide attempt. This could be attributed to males having higher hazards of AF and suicide [51]. So as to properly manage and intervene with side effects, it is crucial to consider age and gender differences into account when evaluating drug safety. Females and the older adults should also pay special attention to the reasons for roflumilast discontinuation.

Psoriasis has been approved for topical treatment with roflumilast cream [36]. Clinical trials treating oral roflumilast for psoriasis have been shown equally excellent safety and tolerability. Similarly to the analysis of a randomized controlled trial, our study thought that headache was the most common AEs in the topical group. It was worth noting that decreased weight was more prevalent in the oral roflumilast group [2, 31]. In phase II clinical trials, topical roflumilast caused minimal weight change in patients with psoriasis [52]. Studies have shown that roflumilast may help obese patients decrease weight by inhibiting adipogenesis and promoting lipolysis through AMPKα activation [53]. A multicenter study showed that patients with low body mass index (BMI <23 kg/m^2^) were more likely to experience adverse reactions and then choose to discontinue treatment with roflumilast [13]. Therefore, patients with BMI <23 kg/m^2^ must be cautious with roflumilast or even avoid choosing an oral dosage form. But if applying the topical formulation of roflumilast, concern should be taken to deal with headache, drug ineffective and dermatologic manifestations that haven’t yet been listed on the label (e.g., burning sensation, exfoliation, and discoloration).

For TTO analysis, only 16.7% reports of roflumilast-related reports in the FAERS database were usable. Based on the available TTO data, 60.1% reports occurred within the first month after initiating therapy. Previous studies similarly found that AEs associated with oral roflumilast occur in the first 4 weeks, whereas roflumilast cream-associated AEs were usually reported in the first 2 weeks after treatment [12, 54]. Long-acting bronchodilators such as long-acting β_2_-agonists and long-acting muscarinic antagonists, which together with inhaled corticosteroid are the basic drugs for long-term management of COPD. Whether used as monotherapy or combination therapy (e.g., dual or triple therapy), roflumilast has demonstrated the greatest benefit in patients with moderate-to-severe COPD [11, 55]. More efficiently than either medication alone, formoterol and roflumilast combination prevented LPS-induced production of TNFα and chemokines that recruited monocytes and T cells in human bronchus model *in vitro* [56]. Finally, we performed sensitivity analyses to identify side effects influenced by roflumilast alone, especially gastrointestinal issues, psychological disorders, pulmonary conditions and heart problems. These AEs may challenge patients’ medication adherence and compromise the effectiveness of roflumilast.

This research is the most comprehensive in-depth, and detailed pharmacovigilance study of roflumilast-related AEs depending on two databases. Nevertheless, it is crucial to acknowledge the limitations of this study. First, the databases have inherent limitations, such as under-reporting, reporting bias, and incomplete data. Under-reporting may occur due to patients’ lack of reporting awareness or clinicians’ failure to identify events. Reporting bias can stem from the inconsistent understanding of reporters, obstructing an accurate evaluation of roflumilast’s safety profile. Additionally, incomplete report details may reduce representativeness and introduce selection bias, which necessitate cautious interpretation of findings like TTO. Second, there are confounding variables in the study. Although this study only selected primary suspected drugs for analysis and conducted sensitivity analyses to exclude concomitant medications, potential confounding variables (e.g., duration of use, comorbidities, demographic variations) may affect the accuracy of the results and hinder stratified assessments based on patient characteristics. Third, signal detection in pharmacovigilance primarily provides associative information, estimating signal strength rather than establishing a definitive causal relationship between roflumilast and AEs. Confirming causality requires further real-world validation, including large-scale prospective cohort studies, electronic health record-based case-control studies, and mechanism-based research (animal models and cell experiments) to verify causality and clarify biological mechanisms. Despite the above limitations, the cross-validation approach using the FAERS and CVARD databases still provides valuable insights and guidance for the post-marketing safety monitoring and rare signal detection of roflumilast.

## Conclusion

This study conducted a comprehensive analysis of roflumilast-associated AEs reported in the FAERS and CVARD databases. Our analysis identified disproportional reporting of several labeled risks while detecting novel safety concerns. Valuable risk suggestions were provided for different populations through subgroup analysis. The findings emphasize that individualized evaluation of patient characteristics and comorbidities before prescribing roflumilast is a key strategy for risk mitigation and rational pharmacotherapy.

## Data Availability

The original contributions presented in the study are included in the article/Supplementary Material, further inquiries can be directed to the corresponding authors.
