# Supplementary material for: Real-world safety profile of roflumilast: a pharmacovigilance analysis using FDA adverse event reporting system and Canada vigilance database
Source: J Pharm Pharm Sci. 2026 Jan 12;28:15678. doi: 10.3389/jpps.2025.15678 (PMC12832561; doi:10.3389/jpps.2025.15678)
Supplement: Supplementary file 1 [file Supplementaryfile1.docx]

Supplementary Material

# Supplementary Figures

**
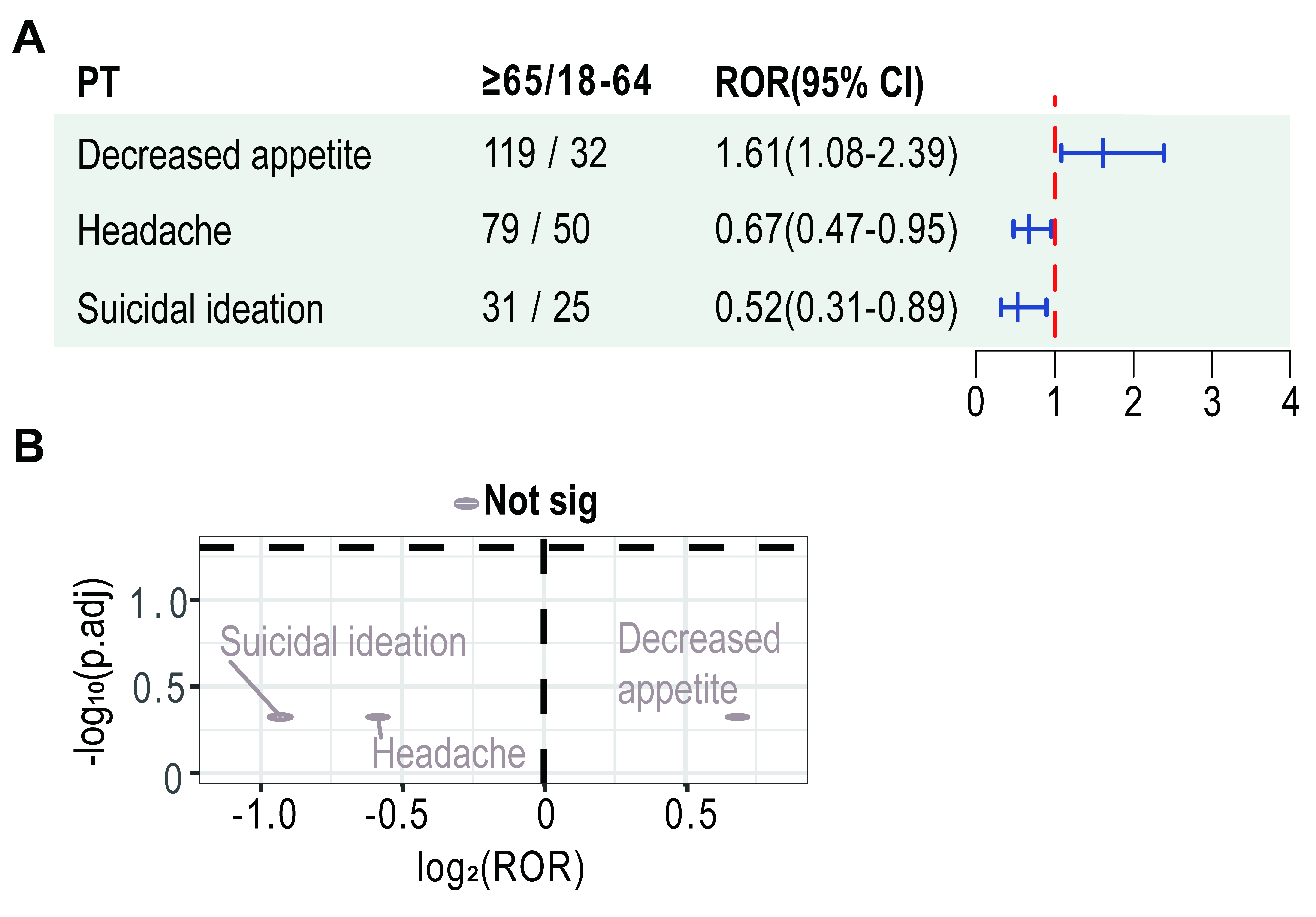
**

**Supplementary Figure S1** The risk differences in the top 50 signals of roflumilast in age subgroup. **(A)** The forest plot of adjusted ROR for age-related AEs. **(B)** Age-differentiated risk signals volcano plot for roflumilast. SOC, System Organ Class; PT, Preferred Term; ROR, Reporting Odds Ratio; CI, confidence interval.


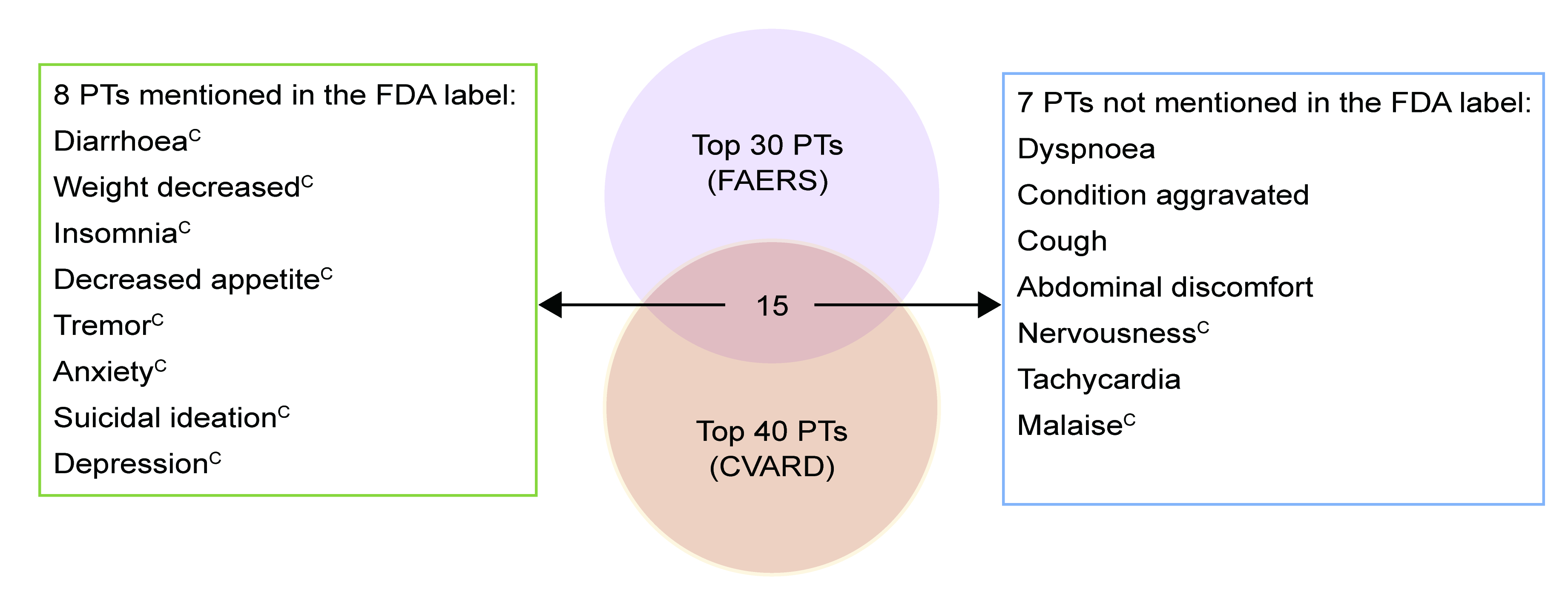


**Supplementary Figure S2** Venn diagram for roflumilast-related side effects showing the overlap of top 30 most frequent positive signals in FAERS and top 40 most frequent positive signals in CVARD. FAERS, FDA Adverse Event Reporting System; CVARD, Canada Vigilance Adverse Reaction Database; PT, Preferred Term.

Alphabets(^C^) indicates AEs documented in the Canadian Product Monographs.

# Supplementary Tables

**Supplementary Table S1:** Two-by-two contingency table for disproportionality analyses.

|  | Target AEs | Other AEs | Total |
| --- | --- | --- | --- |
| Target drug | a | b | a+b |
| Other drugs | c | d | c+d |
| Total | a+c | b+d | a+b+c+d |

Abbreviation: AEs, adverse events; a, number of reports containing both the target drug and target adverse drug reaction; b, number of reports containing other adverse drug reaction of the target drug; c, number of reports containing the target adverse drug reaction of other drugs; d, number of reports containing other drugs and other adverse drug reactions.

**Supplementary Table S2:** Four major algorithms used for signal detection.

| Algorithms | Equation | Criteria |
| --- | --- | --- |
| ROR | ROR = $\frac{a/c}{b/d}$ | lower limit of 95% CI>1, N≥3 |
|  | 95% CI = e^ln(ROR)±1.96(1/a+1/b+1/c+1/d)^0.5^ |  |
| PRR | PRR = $\frac{a\left( c+ⅆ \right)}{c\left( a+b \right)}$ | PRR≥2, χ^2^≥4, N≥3 |
|  | χ^2^ = [(ad-bc)^2](a+b+c+d)/[(a+b)(c+d)(a+c)(b+d)] |  |
| BCPNN | IC = log_2_$\frac{p\left( x,y \right)}{p\left( x \right)p\left( y \right)}$= log_2_$\frac{a\left( a+b+c+ⅆ \right)}{\left( a+b \right)\left( a+c \right)}$ | IC025>0 |
|  | E(IC) = log_2_$\frac{\left( \alpha+\gamma11 \right)\left( a+b+c+ⅆ+\alpha\right)\left( a+b+c+ⅆ+\beta\right)}{\left( a+b+c+ⅆ+\gamma\right)\left( a+b+\alpha1 \right)\left( a+c+\beta1 \right)}$ |  |
|  | V(IC) = $\frac{1}{\left( ln2 \right)^{2}}\{[\frac{\left( a+b+c+ⅆ)-\alpha+\gamma-\gamma11 \right.}{\left( a+\gamma11 \right)\left( 1+a+b+c+ⅆ+\gamma\right)}$] + $[\frac{\left( a+b+c+ⅆ)-\left( a+b \right)+\alpha-\alpha1 \right.}{\left( a+b+\alpha1 \right)\left( 1+a+b+c+ⅆ+\alpha\right)}$] + $[\frac{\left( a+b+c+ⅆ)-\left( a+c \right)+\beta-\beta1 \right.}{\left( a+c+\beta1 \right)\left( 1+a+b+c+ⅆ+\beta\right)}$]} | |
|  | $\gamma$ = $\gamma11 \frac{\left( a+b+c+ⅆ+\alpha\right)\left( a+b+c+ⅆ+\beta\right)}{\left( a+b+\alpha1 \right)\left( a+c+\beta1 \right)}$ |  |
|  | IC-2SD = E(IC) - 2V(IC)^0.5 |  |
|  | α1 = β1 = 1；α = β = 2；γ11 = 1 |  |
| MGPS | EBGM = $\frac{a\left( a+b+c+ⅆ \right)}{\left( a+b \right)\left( a+c \right)}$ | EBGM05>2 |
|  | 95% CI = e^ln(EBGM)±1.96(1/a+1/b+1/c+1/d)^0.5^ |  |

Notes: Equation: a, number of reports containing both the target drug and target adverse drug reaction; b, number of reports containing other adverse drug reaction of the target drug; c, number of reports containing the target adverse drug reaction of other drugs; d, number of reports containing other drugs and other adverse drug reactions. The MGPS employs an empirical Bayesian approach, whereby a prior distribution is obtained by maximum likelihood estimates, and the prior and likelihood are subsequently combined to obtain a posterior distribution.

Abbreviation: 95% CI, 95% confidence interval; N, the number of reports; χ^2^, chi-squared; IC, information component; IC025, the lower limit of 95% CI of the IC; E(IC), the IC expectations; V(IC), the variance of IC; SD, standard deviation; EBGM, empirical Bayesian geometric mean; EBGM05, the lower limit of 95% CI of EBGM.

**Supplementary Table S3:** Two-by-two contingency table for subgroup analyses.

| Roflumilast | Target AEs | Other AEs | Total |
| --- | --- | --- | --- |
| Target group (Female/≥65 years/oral) | a | b | a+b |
| Control group (Male/18-64 years/topical) | c | d | c+d |
| Total | a+c | b+d | a+b+c+d |

Abbreviation: AEs, adverse events; a, number of reports containing target roflumilast's adverse reaction of the target group; b, number of reports containing other roflumilast's adverse reaction of the target group; c, number of reports containing the target adverse drug reaction of control group; d, number of reports containing other roflumilast's adverse reaction of the control group.

**Supplementary Table S4:** Top 100 most frequent adverse events for roflumilast at the PT level from FAERS data.

| PT | Case numbers | ROR (95%Cl) | PRR (χ^2^) | EBGM (EBGM05) | IC (IC025) |
| --- | --- | --- | --- | --- | --- |
| Diarrhea^*^ | 423 | 4.92(4.46-5.43) ^*^ | 4.73(1255.28) ^*^ | 4.72(4.35) ^*^ | 2.24(2.1) ^*^ |
| Weight decreased^*^ | 315 | 8.26(7.38-9.25) ^*^ | 7.99(1934.23) ^*^ | 7.99(7.27) ^*^ | 3(2.83) ^*^ |
| Nausea^*^ | 296 | 2.74(2.44-3.08) ^*^ | 2.68(315.39) ^*^ | 2.68(2.43) ^*^ | 1.42(1.25) ^*^ |
| Dyspnea^*^ | 283 | 3.63(3.23-4.09) ^*^ | 3.54(521.64) ^*^ | 3.54(3.21) ^*^ | 1.83(1.65) ^*^ |
| Insomnia^*^ | 248 | 6.68(5.89-7.58) ^*^ | 6.51(1161.65) ^*^ | 6.51(5.86) ^*^ | 2.7(2.52) ^*^ |
| Headache^*^ | 217 | 2.49(2.17-2.85) ^*^ | 2.45(188.11) ^*^ | 2.45(2.19) ^*^ | 1.29(1.09) ^*^ |
| Decreased appetite^*^ | 208 | 6.62(5.77-7.6) ^*^ | 6.49(967.91) ^*^ | 6.48(5.78) ^*^ | 2.7(2.49) ^*^ |
| Dizziness^*^ | 166 | 2.39(2.05-2.79) ^*^ | 2.36(131.41) ^*^ | 2.36(2.08) ^*^ | 1.24(1.01) ^*^ |
| Tremor^*^ | 119 | 5.07(4.23-6.07) ^*^ | 5.01(382.66) ^*^ | 5.01(4.3) ^*^ | 2.32(2.06) ^*^ |
| Back pain^*^ | 115 | 3.51(2.92-4.23) ^*^ | 3.48(204.02) ^*^ | 3.48(2.98) ^*^ | 1.8(1.53) ^*^ |
| Anxiety^*^ | 110 | 2.72(2.25-3.28) ^*^ | 2.69(117.75) ^*^ | 2.69(2.3) ^*^ | 1.43(1.15) ^*^ |
| Malaise^*^ | 109 | 1.74(1.44-2.1) ^*^ | 1.73(33.84) | 1.73(1.48) | 0.79(0.51) ^*^ |
| Suicidal ideation^*^ | 99 | 7.69(6.31-9.38) ^*^ | 7.61(568.78) ^*^ | 7.6(6.44) ^*^ | 2.93(2.64) ^*^ |
| Asthenia^*^ | 88 | 1.66(1.35-2.05) ^*^ | 1.65(22.89) | 1.65(1.39) | 0.73(0.42) ^*^ |
| Depression^*^ | 84 | 2.56(2.06-3.17) ^*^ | 2.54(78.88) ^*^ | 2.54(2.12) ^*^ | 1.35(1.03) ^*^ |
| Fatigue | 76 | 0.69(0.55-0.87) | 0.7(10.09) | 0.7(0.58) | -0.52(-0.85) |
| Condition aggravated^*^ | 76 | 1.87(1.49-2.34) ^*^ | 1.86(30.24) | 1.86(1.54) | 0.89(0.56) ^*^ |
| Cough^*^ | 70 | 1.81(1.43-2.29) ^*^ | 1.8(25.03) | 1.8(1.48) | 0.85(0.5) ^*^ |
| Feeling abnormal^*^ | 69 | 1.99(1.57-2.52) ^*^ | 1.98(33.68) | 1.98(1.63) | 0.99(0.64) ^*^ |
| Vomiting | 67 | 1.03(0.81-1.3) | 1.03(0.04) | 1.03(0.84) | 0.04(-0.31) |
| Drug ineffective | 65 | 0.35(0.27-0.44) | 0.35(79.95) | 0.35(0.29) | -1.51(-1.87) |
| Abdominal pain upper^*^ | 57 | 2(1.54-2.59) ^*^ | 1.99(28.27) | 1.99(1.6) | 0.99(0.61) ^*^ |
| Influenza like illness^*^ | 55 | 4.64(3.56-6.05) ^*^ | 4.62(155.99) ^*^ | 4.62(3.7) ^*^ | 2.21(1.82) ^*^ |
| Muscle spasms^*^ | 47 | 1.81(1.36-2.4) ^*^ | 1.8(16.79) | 1.8(1.42) | 0.85(0.43) ^*^ |
| Abdominal discomfort^*^ | 46 | 1.98(1.48-2.64) ^*^ | 1.97(22.15) | 1.97(1.55) | 0.98(0.56) ^*^ |
| Heart rate increased^*^ | 46 | 3.31(2.48-4.43) ^*^ | 3.3(73.81) ^*^ | 3.3(2.59) ^*^ | 1.72(1.3) ^*^ |
| Pain | 41 | 0.46(0.34-0.63) | 0.46(25.5) | 0.47(0.36) | -1.1(-1.55) |
| Abdominal pain | 40 | 1.23(0.9-1.67) | 1.23(1.67) | 1.23(0.95) | 0.29(-0.16) |
| Chest pain^*^ | 39 | 1.47(1.07-2.01) ^*^ | 1.47(5.83) | 1.47(1.13) | 0.55(0.1) ^*^ |
| Nervousness^*^ | 37 | 4.85(3.51-6.69) ^*^ | 4.83(112.33) ^*^ | 4.83(3.68) ^*^ | 2.27(1.8) ^*^ |
| Pruritus | 37 | 0.74(0.54-1.02) | 0.74(3.39) | 0.74(0.56) | -0.43(-0.9) |
| Pain in extremity | 36 | 0.84(0.61-1.17) | 0.85(1.02) | 0.85(0.64) | -0.24(-0.72) |
| Myalgia^*^ | 35 | 1.46(1.04-2.03) ^*^ | 1.45(4.98) | 1.45(1.1) | 0.54(0.06) ^*^ |
| Palpitations^*^ | 35 | 2.13(1.53-2.97) ^*^ | 2.12(20.82) ^*^ | 2.12(1.61) | 1.09(0.6) ^*^ |
| Product use issue | 34 | 1.33(0.95-1.87) | 1.33(2.85) | 1.33(1.01) | 0.42(-0.07) |
| Atrial fibrillation^*^ | 32 | 2.32(1.64-3.28) ^*^ | 2.31(23.87) ^*^ | 2.31(1.73) | 1.21(0.71) ^*^ |
| Rash | 31 | 0.52(0.36-0.74) | 0.52(13.88) | 0.52(0.39) | -0.95(-1.46) |
| Sleep disorder^*^ | 30 | 3.13(2.19-4.48) ^*^ | 3.12(43.28) ^*^ | 3.12(2.31) ^*^ | 1.64(1.12) ^*^ |
| Tachycardia^*^ | 28 | 2.24(1.55-3.25) ^*^ | 2.24(19.15) ^*^ | 2.24(1.64) | 1.16(0.62) ^*^ |
| Pyrexia | 27 | 0.55(0.37-0.8) | 0.55(10.19) | 0.55(0.4) | -0.87(-1.42) |
| Gastrointestinal disorder^*^ | 27 | 2.33(1.6-3.41) ^*^ | 2.33(20.5) ^*^ | 2.33(1.7) | 1.22(0.67) ^*^ |
| Arthralgia | 26 | 0.45(0.3-0.66) | 0.45(17.66) | 0.45(0.33) | -1.15(-1.71) |
| Product dose omission issue | 25 | 0.77(0.52-1.14) | 0.77(1.72) | 0.77(0.55) | -0.38(-0.94) |
| Dehydration | 23 | 1.21(0.81-1.83) | 1.21(0.85) | 1.21(0.86) | 0.28(-0.31) |
| Respiratory failure^*^ | 23 | 2.22(1.47-3.34) ^*^ | 2.21(15.3) ^*^ | 2.21(1.57) | 1.15(0.56) ^*^ |
| Panic attack^*^ | 22 | 4.26(2.8-6.48) ^*^ | 4.25(54.75) ^*^ | 4.25(3) ^*^ | 2.09(1.49) ^*^ |
| Chest discomfort^*^ | 22 | 1.55(1.02-2.36) ^*^ | 1.55(4.29) | 1.55(1.09) | 0.63(0.03) ^*^ |
| Lung disorder^*^ | 21 | 3.08(2.01-4.73) ^*^ | 3.08(29.49) ^*^ | 3.08(2.15) ^*^ | 1.62(1.01) ^*^ |
| Productive cough^*^ | 21 | 3.27(2.13-5.01) ^*^ | 3.26(32.95) ^*^ | 3.26(2.28) ^*^ | 1.71(1.09) ^*^ |
| Dyspepsia | 20 | 1.47(0.95-2.28) | 1.47(3) | 1.47(1.02) | 0.55(-0.08) |
| Blood pressure increased | 20 | 0.92(0.59-1.43) | 0.92(0.14) | 0.92(0.64) | -0.12(-0.75) |
| Depressed mood^*^ | 20 | 2.75(1.77-4.26) ^*^ | 2.75(22.2) ^*^ | 2.74(1.9) | 1.46(0.83) ^*^ |
| Wheezing^*^ | 20 | 2.5(1.61-3.88) ^*^ | 2.5(17.98) ^*^ | 2.5(1.73) | 1.32(0.69) ^*^ |
| Vision blurred | 20 | 1.05(0.68-1.63) | 1.05(0.05) | 1.05(0.73) | 0.07(-0.56) |
| Memory impairment | 20 | 1.02(0.66-1.58) | 1.02(0.01) | 1.02(0.71) | 0.03(-0.6) |
| Hallucination^*^ | 20 | 1.92(1.24-2.98) ^*^ | 1.92(8.8) | 1.92(1.33) | 0.94(0.31) ^*^ |
| Myocardial infarction | 19 | 0.73(0.47-1.15) | 0.73(1.88) | 0.73(0.5) | -0.45(-1.1) |
| Nightmare^*^ | 19 | 3.82(2.44-6) ^*^ | 3.82(39.49) ^*^ | 3.81(2.62) ^*^ | 1.93(1.29) ^*^ |
| Suicide attempt^*^ | 19 | 2.21(1.41-3.47) ^*^ | 2.21(12.58) ^*^ | 2.21(1.52) | 1.14(0.5) ^*^ |
| Confusional state | 18 | 0.78(0.49-1.24) | 0.78(1.11) | 0.78(0.53) | -0.36(-1.02) |
| Restlessness^*^ | 17 | 3.25(2.02-5.23) ^*^ | 3.25(26.45) ^*^ | 3.25(2.18) ^*^ | 1.7(1.02) ^*^ |
| Hypotension | 17 | 0.6(0.37-0.96) | 0.6(4.58) | 0.6(0.4) | -0.74(-1.42) |
| Lung neoplasm malignant^*^ | 17 | 2.82(1.75-4.54) ^*^ | 2.82(19.91) ^*^ | 2.81(1.89) | 1.49(0.81) ^*^ |
| Oedema peripheral | 17 | 0.96(0.59-1.54) | 0.96(0.03) | 0.96(0.64) | -0.06(-0.74) |
| Drug intolerance | 17 | 1.28(0.79-2.05) | 1.28(1.01) | 1.28(0.86) | 0.35(-0.33) |
| Visual impairment | 16 | 0.95(0.58-1.55) | 0.95(0.05) | 0.95(0.63) | -0.08(-0.78) |
| Crying^*^ | 16 | 3.01(1.84-4.91) ^*^ | 3(21.38) ^*^ | 3(1.99) | 1.59(0.89) ^*^ |
| Rhinorrhea^*^ | 16 | 1.8(1.1-2.94) ^*^ | 1.8(5.67) | 1.8(1.19) | 0.85(0.15) ^*^ |
| Gastroesophageal reflux disease | 16 | 1.44(0.88-2.35) | 1.44(2.12) | 1.44(0.95) | 0.52(-0.18) |
| Influenza | 16 | 1.08(0.66-1.76) | 1.08(0.08) | 1.08(0.71) | 0.1(-0.6) |
| Gait disturbance | 16 | 0.58(0.36-0.95) | 0.58(4.84) | 0.58(0.39) | -0.78(-1.48) |
| Chills | 16 | 0.96(0.59-1.56) | 0.96(0.03) | 0.96(0.63) | -0.07(-0.77) |
| Skin burning sensation | 16 | 1.6(0.98-2.61) | 1.6(3.57) | 1.6(1.06) | 0.67(-0.03) |
| Abdominal distension | 15 | 1.04(0.63-1.73) | 1.04(0.02) | 1.04(0.68) | 0.06(-0.67) |
| Muscular weakness | 15 | 0.93(0.56-1.54) | 0.93(0.08) | 0.93(0.61) | -0.1(-0.83) |
| Hyperhidrosis | 15 | 0.81(0.49-1.35) | 0.81(0.66) | 0.81(0.53) | -0.3(-1.02) |
| Drug interaction | 15 | 0.66(0.4-1.1) | 0.66(2.54) | 0.66(0.44) | -0.59(-1.31) |
| Fall | 15 | 0.32(0.19-0.53) | 0.32(21.94) | 0.32(0.21) | -1.65(-2.37) |
| Feeling jittery^*^ | 15 | 5.46(3.29-9.05) ^*^ | 5.45(54.43) ^*^ | 5.44(3.56) ^*^ | 2.44(1.72) ^*^ |
| Mood altered^*^ | 15 | 3.94(2.37-6.54) ^*^ | 3.93(32.8) ^*^ | 3.93(2.57) ^*^ | 1.97(1.25) ^*^ |
| Paresthesia | 15 | 0.66(0.4-1.1) | 0.66(2.59) | 0.66(0.43) | -0.59(-1.32) |
| Nasopharyngitis | 14 | 0.55(0.32-0.92) | 0.55(5.28) | 0.55(0.35) | -0.87(-1.62) |
| Cardiac disorder | 14 | 1.04(0.62-1.76) | 1.04(0.02) | 1.04(0.67) | 0.06(-0.69) |
| Hypersensitivity | 14 | 0.54(0.32-0.9) | 0.54(5.64) | 0.54(0.35) | -0.9(-1.64) |
| Constipation | 14 | 0.47(0.28-0.8) | 0.47(8.17) | 0.47(0.31) | -1.08(-1.82) |
| Agitation | 14 | 1.32(0.78-2.23) | 1.32(1.09) | 1.32(0.85) | 0.4(-0.34) |
| Erythema | 14 | 0.48(0.29-0.82) | 0.48(7.72) | 0.48(0.31) | -1.05(-1.79) |
| Skin exfoliation | 14 | 1.23(0.73-2.08) | 1.23(0.61) | 1.23(0.79) | 0.3(-0.44) |
| Syncope | 13 | 0.91(0.53-1.57) | 0.91(0.12) | 0.91(0.58) | -0.14(-0.91) |
| Anemia | 13 | 0.47(0.28-0.82) | 0.48(7.54) | 0.48(0.3) | -1.07(-1.84) |
| Urticaria | 13 | 0.57(0.33-0.98) | 0.57(4.25) | 0.57(0.36) | -0.81(-1.58) |
| Somnolence | 13 | 0.46(0.27-0.79) | 0.46(8.38) | 0.46(0.29) | -1.13(-1.9) |
| Cerebrovascular accident | 13 | 0.53(0.31-0.91) | 0.53(5.44) | 0.53(0.34) | -0.92(-1.69) |
| Urinary tract infection | 13 | 0.54(0.32-0.94) | 0.55(4.94) | 0.55(0.35) | -0.87(-1.65) |
| Pollakiuria^*^ | 13 | 2.23(1.29-3.84) ^*^ | 2.23(8.77) ^*^ | 2.22(1.41) | 1.15(0.38) ^*^ |
| Frequent bowel movements^*^ | 13 | 3.62(2.1-6.24) ^*^ | 3.62(24.6) ^*^ | 3.61(2.29) ^*^ | 1.85(1.08) ^*^ |
| Hematochezia | 12 | 1.58(0.9-2.79) | 1.58(2.57) | 1.58(0.98) | 0.66(-0.14) |
| Epistaxis | 12 | 1.13(0.64-1.98) | 1.13(0.17) | 1.13(0.7) | 0.17(-0.63) |
| Cardiac arrest | 12 | 1.02(0.58-1.79) | 1.02(0) | 1.02(0.63) | 0.02(-0.78) |
| Neoplasm malignant | 12 | 1.29(0.73-2.28) | 1.29(0.8) | 1.29(0.8) | 0.37(-0.43) |

Asterisks (^*^) indicate statistically significant signals in algorithm. ROR, Reporting Odds Ratio; PRR, Proportional Reporting Ratio; EBGM, Empirical Bayesian Geometric Mean; EBGM05, the lower limit of the 95% CI of EBGM; IC, information component; IC025, the lower limit of the 95% CI of the IC; CI, confidence interval; PT, Preferred Term.

**Supplementary Table S5:** Top 50 most frequent adverse events for roflumilast at the PT level in males from FAERS data.

| PT | Case numbers | ROR (95%Cl) | PRR (χ^2^) | EBGM (EBGM05) | IC (IC025) |
| --- | --- | --- | --- | --- | --- |
| Diarrhea^*^ | 175 | 4.71(4.05-5.48) ^*^ | 4.54(487.16) ^*^ | 4.53(3.99) ^*^ | 2.18(1.96) ^*^ |
| Dyspnea^*^ | 139 | 4(3.38-4.74) ^*^ | 3.89(301.47) ^*^ | 3.89(3.38) ^*^ | 1.96(1.71) ^*^ |
| Weight decreased^*^ | 137 | 7.17(6.05-8.51) ^*^ | 6.95(700.48) ^*^ | 6.94(6.02) ^*^ | 2.8(2.54) ^*^ |
| Insomnia^*^ | 106 | 6.76(5.57-8.2) ^*^ | 6.6(504.73) ^*^ | 6.59(5.6) ^*^ | 2.72(2.44) ^*^ |
| Decreased appetite^*^ | 100 | 6.42(5.27-7.84) ^*^ | 6.28(445.27) ^*^ | 6.27(5.31) ^*^ | 2.65(2.36) ^*^ |
| Nausea^*^ | 97 | 2.71(2.21-3.31) ^*^ | 2.66(101.63) ^*^ | 2.66(2.25) ^*^ | 1.41(1.12) ^*^ |
| Headache^*^ | 71 | 2.47(1.95-3.12) ^*^ | 2.44(60.69) ^*^ | 2.44(2) | 1.29(0.94) ^*^ |
| Dizziness^*^ | 67 | 2.4(1.88-3.05) ^*^ | 2.37(53.47) ^*^ | 2.37(1.94) | 1.24(0.89) ^*^ |
| Suicidal ideation^*^ | 54 | 8.71(6.66-11.4) ^*^ | 8.6(362.61) ^*^ | 8.59(6.86) ^*^ | 3.1(2.71) ^*^ |
| Anxiety^*^ | 50 | 3.16(2.39-4.17) ^*^ | 3.13(72.69) ^*^ | 3.13(2.48) ^*^ | 1.65(1.24) ^*^ |
| Tremor^*^ | 48 | 4.9(3.68-6.51) ^*^ | 4.85(146.87) ^*^ | 4.84(3.82) ^*^ | 2.28(1.86) ^*^ |
| Depression^*^ | 46 | 3.3(2.47-4.41) ^*^ | 3.27(72.8) ^*^ | 3.27(2.56) ^*^ | 1.71(1.29) ^*^ |
| Malaise^*^ | 45 | 1.93(1.44-2.58) ^*^ | 1.92(19.8) | 1.92(1.5) | 0.94(0.51) ^*^ |
| Asthenia^*^ | 42 | 1.67(1.23-2.26) ^*^ | 1.66(11.18) | 1.66(1.29) | 0.73(0.29) ^*^ |
| Condition aggravated^*^ | 41 | 2.38(1.75-3.24) ^*^ | 2.37(32.46) ^*^ | 2.37(1.83) | 1.24(0.79) ^*^ |
| Back pain^*^ | 36 | 2.78(2-3.86) ^*^ | 2.76(40.52) ^*^ | 2.76(2.1) ^*^ | 1.46(0.99) ^*^ |
| Fatigue | 35 | 0.78(0.56-1.08) | 0.78(2.21) | 0.78(0.59) | -0.36(-0.84) |
| Cough^*^ | 32 | 1.99(1.4-2.81) ^*^ | 1.98(15.54) | 1.98(1.48) | 0.98(0.48) ^*^ |
| Feeling abnormal^*^ | 31 | 2.46(1.73-3.5) ^*^ | 2.44(26.54) ^*^ | 2.44(1.82) | 1.29(0.78) ^*^ |
| Drug ineffective | 27 | 0.35(0.24-0.51) | 0.35(32.34) | 0.35(0.26) | -1.49(-2.04) |
| Muscle spasms^*^ | 24 | 2.49(1.66-3.71) ^*^ | 2.48(21.16) ^*^ | 2.48(1.77) | 1.31(0.73) ^*^ |
| Vomiting | 24 | 0.99(0.67-1.48) | 0.99(0) | 0.99(0.71) | -0.01(-0.59) |
| Atrial fibrillation^*^ | 22 | 2.63(1.73-4) ^*^ | 2.62(22.13) ^*^ | 2.62(1.85) | 1.39(0.79) ^*^ |
| Heart rate increased^*^ | 21 | 3.88(2.53-5.96) ^*^ | 3.86(44.61) ^*^ | 3.86(2.7) ^*^ | 1.95(1.33) ^*^ |
| Abdominal pain | 20 | 1.52(0.98-2.36) | 1.52(3.56) | 1.52(1.05) | 0.6(-0.03) |
| Chest pain^*^ | 20 | 1.65(1.07-2.57) ^*^ | 1.65(5.14) | 1.65(1.14) | 0.72(0.09) ^*^ |
| Nervousness^*^ | 20 | 7.91(5.1-12.28) ^*^ | 7.87(119.91) ^*^ | 7.86(5.44) ^*^ | 2.98(2.34) ^*^ |
| Influenza like illness^*^ | 19 | 4.27(2.72-6.71) ^*^ | 4.26(47.33) ^*^ | 4.25(2.92) ^*^ | 2.09(1.44) ^*^ |
| Myalgia^*^ | 18 | 1.72(1.08-2.73) ^*^ | 1.72(5.41) | 1.72(1.17) | 0.78(0.12) ^*^ |
| Abdominal discomfort^*^ | 18 | 2.07(1.3-3.29) ^*^ | 2.06(9.89) ^*^ | 2.06(1.4) | 1.04(0.38) ^*^ |
| Pyrexia | 18 | 0.71(0.45-1.13) | 0.71(2.1) | 0.71(0.48) | -0.49(-1.15) |
| Sleep disorder^*^ | 18 | 4.27(2.69-6.79) ^*^ | 4.25(44.82) ^*^ | 4.25(2.89) ^*^ | 2.09(1.42) ^*^ |
| Pain in extremity | 17 | 1.13(0.7-1.82) | 1.13(0.26) | 1.13(0.76) | 0.18(-0.5) |
| Abdominal pain upper | 16 | 1.55(0.95-2.53) | 1.54(3.08) | 1.54(1.02) | 0.63(-0.07) |
| Dyspepsia^*^ | 14 | 2.74(1.62-4.63) ^*^ | 2.73(15.37) ^*^ | 2.73(1.76) | 1.45(0.7) ^*^ |
| Arthralgia | 14 | 0.68(0.4-1.15) | 0.68(2.05) | 0.68(0.44) | -0.55(-1.29) |
| Tachycardia^*^ | 13 | 2.21(1.28-3.81) ^*^ | 2.21(8.59) ^*^ | 2.21(1.4) | 1.14(0.37) ^*^ |
| Productive cough^*^ | 13 | 4.12(2.39-7.11) ^*^ | 4.11(30.59) ^*^ | 4.11(2.6) ^*^ | 2.04(1.27) ^*^ |
| Product use issue | 13 | 1.22(0.71-2.11) | 1.22(0.53) | 1.22(0.78) | 0.29(-0.48) |
| Suicide attempt^*^ | 13 | 3.22(1.87-5.56) ^*^ | 3.21(19.85) ^*^ | 3.21(2.04) ^*^ | 1.68(0.91) ^*^ |
| Oedema peripheral | 12 | 1.48(0.84-2.62) | 1.48(1.89) | 1.48(0.92) | 0.57(-0.23) |
| Rash | 12 | 0.48(0.27-0.84) | 0.48(6.81) | 0.48(0.3) | -1.06(-1.86) |
| Dehydration | 11 | 1.16(0.64-2.1) | 1.16(0.25) | 1.16(0.71) | 0.22(-0.62) |
| Confusional state | 11 | 0.93(0.51-1.67) | 0.93(0.06) | 0.93(0.56) | -0.11(-0.95) |
| Respiratory failure | 11 | 1.71(0.95-3.09) | 1.71(3.24) | 1.71(1.04) | 0.77(-0.06) |
| Chest discomfort^*^ | 11 | 2.04(1.13-3.69) ^*^ | 2.04(5.83) ^*^ | 2.04(1.24) | 1.03(0.19) ^*^ |
| Hallucination^*^ | 11 | 1.8(1-3.26) | 1.8(3.91) | 1.8(1.1) | 0.85(0.01) ^*^ |
| Restlessness^*^ | 10 | 3.52(1.89-6.55) ^*^ | 3.52(18.01) ^*^ | 3.51(2.09) ^*^ | 1.81(0.94) ^*^ |
| Hypotension | 10 | 0.64(0.35-1.2) | 0.64(1.97) | 0.64(0.38) | -0.63(-1.5) |
| Palpitations^*^ | 10 | 2.01(1.08-3.74) ^*^ | 2.01(5.06) ^*^ | 2.01(1.19) | 1(0.13) ^*^ |

Asterisks (^*^) indicate statistically significant signals in algorithm. ROR, Reporting Odds Ratio; PRR, Proportional Reporting Ratio; EBGM, Empirical Bayesian Geometric Mean; EBGM05, the lower limit of the 95% CI of EBGM; IC, information component; IC025, the lower limit of the 95% CI of the IC; CI, confidence interval; PT, Preferred Term.

**Supplementary Table S6:** Top 50 most frequent adverse events for roflumilast at the PT level in females from FAERS data.

| PT | Case numbers | ROR (95%Cl) | PRR (χ^2^) | EBGM (EBGM05) | IC (IC025) |
| --- | --- | --- | --- | --- | --- |
| Diarrhea^*^ | 220 | 5.2(4.54-5.96) ^*^ | 4.97(705.09) ^*^ | 4.97(4.43) ^*^ | 2.31(2.11) ^*^ |
| Nausea^*^ | 179 | 2.98(2.57-3.46) ^*^ | 2.89(225.28) ^*^ | 2.89(2.55) ^*^ | 1.53(1.31) ^*^ |
| Weight decreased^*^ | 170 | 9.86(8.45-11.5) ^*^ | 9.48(1294.28) ^*^ | 9.47(8.33) ^*^ | 3.24(3.02) ^*^ |
| Dyspnea^*^ | 137 | 3.6(3.03-4.27) ^*^ | 3.51(248.05) ^*^ | 3.51(3.04) ^*^ | 1.81(1.56) ^*^ |
| Insomnia^*^ | 126 | 6.77(5.67-8.08) ^*^ | 6.59(599.29) ^*^ | 6.58(5.67) ^*^ | 2.72(2.46) ^*^ |
| Headache^*^ | 124 | 2.53(2.11-3.02) ^*^ | 2.48(110.95) ^*^ | 2.48(2.14) ^*^ | 1.31(1.05) ^*^ |
| Decreased appetite^*^ | 107 | 7.49(6.18-9.08) ^*^ | 7.32(585.45) ^*^ | 7.31(6.23) ^*^ | 2.87(2.59) ^*^ |
| Dizziness^*^ | 94 | 2.61(2.13-3.2) ^*^ | 2.57(91.2) ^*^ | 2.57(2.17) ^*^ | 1.36(1.06) ^*^ |
| Back pain^*^ | 75 | 4.32(3.44-5.43) ^*^ | 4.26(187.65) ^*^ | 4.26(3.51) ^*^ | 2.09(1.76) ^*^ |
| Tremor^*^ | 66 | 5.51(4.32-7.03) ^*^ | 5.44(239.52) ^*^ | 5.43(4.43) ^*^ | 2.44(2.09) ^*^ |
| Malaise^*^ | 59 | 1.74(1.35-2.26) ^*^ | 1.73(18.44) | 1.73(1.4) | 0.79(0.42) ^*^ |
| Anxiety^*^ | 56 | 2.7(2.08-3.52) ^*^ | 2.68(59.19) ^*^ | 2.68(2.15) ^*^ | 1.42(1.04) ^*^ |
| Asthenia^*^ | 43 | 1.7(1.26-2.29) ^*^ | 1.69(12.14) | 1.69(1.31) | 0.76(0.32) ^*^ |
| Abdominal pain upper^*^ | 41 | 2.64(1.94-3.59) ^*^ | 2.62(41.26) ^*^ | 2.62(2.03) ^*^ | 1.39(0.94) ^*^ |
| Vomiting | 41 | 1.18(0.87-1.6) | 1.18(1.11) | 1.18(0.91) | 0.24(-0.21) |
| Fatigue | 39 | 0.71(0.52-0.97) | 0.71(4.73) | 0.71(0.54) | -0.5(-0.95) |
| Depression^*^ | 37 | 2.19(1.59-3.03) ^*^ | 2.18(23.82) ^*^ | 2.18(1.66) | 1.13(0.66) ^*^ |
| Cough^*^ | 36 | 1.82(1.31-2.53) ^*^ | 1.81(13.2) | 1.81(1.38) | 0.86(0.38) ^*^ |
| Feeling abnormal^*^ | 35 | 1.86(1.33-2.6) ^*^ | 1.85(13.82) | 1.85(1.4) | 0.89(0.41) ^*^ |
| Influenza like illness^*^ | 34 | 5.2(3.71-7.29) ^*^ | 5.16(114.29) ^*^ | 5.16(3.89) ^*^ | 2.37(1.88) ^*^ |
| Suicidal ideation^*^ | 33 | 5.56(3.95-7.83) ^*^ | 5.52(122.33) ^*^ | 5.52(4.14) ^*^ | 2.46(1.97) ^*^ |
| Pain | 28 | 0.59(0.41-0.86) | 0.6(7.78) | 0.6(0.44) | -0.75(-1.29) |
| Abdominal discomfort^*^ | 27 | 2.21(1.52-3.23) ^*^ | 2.21(17.86) ^*^ | 2.21(1.61) | 1.14(0.59) ^*^ |
| Condition aggravated | 25 | 1.33(0.9-1.97) | 1.32(1.99) | 1.32(0.95) | 0.41(-0.16) |
| Heart rate increased^*^ | 24 | 3.28(2.19-4.89) ^*^ | 3.26(37.69) ^*^ | 3.26(2.33) ^*^ | 1.71(1.13) ^*^ |
| Drug ineffective | 23 | 0.27(0.18-0.4) | 0.27(45.86) | 0.27(0.19) | -1.88(-2.47) |
| Muscle spasms^*^ | 22 | 1.56(1.03-2.37) ^*^ | 1.56(4.39) | 1.56(1.1) | 0.64(0.03) ^*^ |
| Palpitations^*^ | 20 | 2.09(1.35-3.25) ^*^ | 2.09(11.36) ^*^ | 2.09(1.45) | 1.06(0.43) ^*^ |
| Abdominal pain | 20 | 1.19(0.76-1.84) | 1.19(0.58) | 1.19(0.82) | 0.25(-0.39) |
| Pain in extremity | 19 | 0.79(0.5-1.24) | 0.79(1.09) | 0.79(0.54) | -0.34(-0.99) |
| Pruritus | 19 | 0.73(0.46-1.14) | 0.73(1.91) | 0.73(0.5) | -0.45(-1.1) |
| Product use issue | 17 | 1.43(0.89-2.31) | 1.43(2.21) | 1.43(0.96) | 0.52(-0.17) |
| Nervousness^*^ | 16 | 3.6(2.21-5.89) ^*^ | 3.59(29.96) ^*^ | 3.59(2.38) ^*^ | 1.84(1.14) ^*^ |
| Chest pain | 16 | 1.22(0.75-1.99) | 1.22(0.63) | 1.22(0.81) | 0.29(-0.42) |
| Rash | 15 | 0.51(0.31-0.85) | 0.51(7) | 0.51(0.34) | -0.96(-1.69) |
| Gastrointestinal disorder^*^ | 15 | 2.66(1.6-4.41) ^*^ | 2.65(15.42) ^*^ | 2.65(1.73) | 1.41(0.68) ^*^ |
| Tachycardia^*^ | 15 | 2.57(1.55-4.26) ^*^ | 2.56(14.29) ^*^ | 2.56(1.67) | 1.36(0.63) ^*^ |
| Memory impairment | 15 | 1.4(0.85-2.33) | 1.4(1.74) | 1.4(0.92) | 0.49(-0.23) |
| Lung disorder^*^ | 14 | 4.54(2.68-7.67) ^*^ | 4.52(38.43) ^*^ | 4.52(2.91) ^*^ | 2.18(1.43) ^*^ |
| Panic attack^*^ | 13 | 4.58(2.66-7.9) ^*^ | 4.57(36.29) ^*^ | 4.57(2.9) ^*^ | 2.19(1.42) ^*^ |
| Myalgia | 13 | 1.09(0.63-1.89) | 1.09(0.11) | 1.09(0.69) | 0.13(-0.64) |
| Dehydration | 12 | 1.36(0.77-2.39) | 1.36(1.13) | 1.36(0.84) | 0.44(-0.36) |
| Respiratory failure^*^ | 12 | 3.09(1.75-5.45) ^*^ | 3.09(16.92) ^*^ | 3.08(1.92) | 1.62(0.82) ^*^ |
| Sleep disorder^*^ | 12 | 2.56(1.46-4.52) ^*^ | 2.56(11.42) ^*^ | 2.56(1.59) | 1.36(0.55) ^*^ |
| Depressed mood^*^ | 12 | 3.16(1.79-5.57) ^*^ | 3.15(17.65) ^*^ | 3.15(1.96) | 1.66(0.85) ^*^ |
| Gastroesophageal reflux disease^*^ | 11 | 1.89(1.04-3.41) ^*^ | 1.89(4.58) | 1.89(1.15) | 0.91(0.08) ^*^ |
| Influenza | 11 | 1.36(0.75-2.46) | 1.36(1.06) | 1.36(0.83) | 0.45(-0.39) |
| Wheezing^*^ | 11 | 2.78(1.54-5.02) ^*^ | 2.77(12.48) ^*^ | 2.77(1.69) | 1.47(0.64) ^*^ |
| Chest discomfort | 10 | 1.32(0.71-2.45) | 1.32(0.76) | 1.32(0.78) | 0.4(-0.47) |
| Hyperhidrosis | 10 | 1.15(0.62-2.14) | 1.15(0.2) | 1.15(0.69) | 0.2(-0.67) |

Asterisks (^*^) indicate statistically significant signals in algorithm. ROR, Reporting Odds Ratio; PRR, Proportional Reporting Ratio; EBGM, Empirical Bayesian Geometric Mean; EBGM05, the lower limit of the 95% CI of EBGM; IC, information component; IC025, the lower limit of the 95% CI of the IC; CI, confidence interval; PT, Preferred Term.

**Supplementary Table S7:** Top 50 most frequent adverse events at the PT level for roflumilast in patients aged ≥ 65 from FAERS data.

| PT | Case numbers | ROR (95%Cl) | PRR (χ2) | EBGM (EBGM05) | IC (IC025) |
| --- | --- | --- | --- | --- | --- |
| Diarrhea^*^ | 180 | 4.04(3.48-4.69) ^*^ | 3.88(389.81) ^*^ | 3.88(3.42) ^*^ | 1.96(1.74) ^*^ |
| Weight decreased^*^ | 156 | 8.49(7.23-9.97) ^*^ | 8.15(981.58) ^*^ | 8.13(7.11) ^*^ | 3.02(2.79) ^*^ |
| Nausea^*^ | 135 | 3.28(2.76-3.9) ^*^ | 3.2(205.9) ^*^ | 3.19(2.76) ^*^ | 1.67(1.42) ^*^ |
| Dyspnea^*^ | 133 | 3.21(2.7-3.82) ^*^ | 3.12(194.26) ^*^ | 3.12(2.7) ^*^ | 1.64(1.39) ^*^ |
| Decreased appetite^*^ | 119 | 6.33(5.27-7.6) ^*^ | 6.14(514.51) ^*^ | 6.14(5.26) ^*^ | 2.62(2.35) ^*^ |
| Insomnia^*^ | 112 | 8.64(7.16-10.43) ^*^ | 8.39(730.46) ^*^ | 8.38(7.15) ^*^ | 3.07(2.79) ^*^ |
| Dizziness^*^ | 82 | 2.48(1.99-3.09) ^*^ | 2.45(70.71) ^*^ | 2.44(2.04) ^*^ | 1.29(0.97) ^*^ |
| Headache^*^ | 79 | 3.04(2.43-3.8) ^*^ | 3(105.73) ^*^ | 2.99(2.48) ^*^ | 1.58(1.26) ^*^ |
| Tremor^*^ | 53 | 5.18(3.95-6.8) ^*^ | 5.12(175.85) ^*^ | 5.11(4.07) ^*^ | 2.35(1.96) ^*^ |
| Back pain^*^ | 52 | 3.45(2.62-4.54) ^*^ | 3.41(89.1) ^*^ | 3.41(2.71) ^*^ | 1.77(1.37) ^*^ |
| Asthenia^*^ | 43 | 1.37(1.01-1.85) ^*^ | 1.36(4.18) | 1.36(1.06) | 0.45(0.01) ^*^ |
| Anxiety^*^ | 43 | 4.79(3.55-6.48) ^*^ | 4.75(127.29) ^*^ | 4.74(3.69) ^*^ | 2.25(1.81) ^*^ |
| Malaise^*^ | 39 | 1.42(1.04-1.95) ^*^ | 1.42(4.83) | 1.42(1.09) | 0.5(0.04) ^*^ |
| Fatigue | 38 | 0.85(0.62-1.17) | 0.85(1.03) | 0.85(0.65) | -0.24(-0.7) |
| Depression^*^ | 38 | 5.27(3.83-7.26) ^*^ | 5.22(129.8) ^*^ | 5.22(3.99) ^*^ | 2.38(1.92) ^*^ |
| Cough^*^ | 33 | 1.78(1.26-2.51) ^*^ | 1.77(11.21) | 1.77(1.33) | 0.83(0.33) ^*^ |
| Suicidal ideation^*^ | 31 | 19.5(13.68-27.8) ^*^ | 19.34(536.24) ^*^ | 19.23(14.3) ^*^ | 4.27(3.75) ^*^ |
| Condition aggravated^*^ | 30 | 1.96(1.37-2.81) ^*^ | 1.95(13.98) | 1.95(1.44) | 0.96(0.44) ^*^ |
| Abdominal pain upper^*^ | 27 | 2.36(1.62-3.45) ^*^ | 2.35(21) ^*^ | 2.35(1.71) | 1.23(0.68) ^*^ |
| Drug ineffective | 25 | 0.5(0.34-0.74) | 0.51(12.22) | 0.51(0.36) | -0.98(-1.55) |
| Abdominal pain^*^ | 25 | 2.08(1.4-3.08) ^*^ | 2.07(13.87) ^*^ | 2.07(1.49) | 1.05(0.48) ^*^ |
| Vomiting | 23 | 0.91(0.61-1.38) | 0.91(0.19) | 0.91(0.65) | -0.13(-0.72) |
| Influenza like illness^*^ | 22 | 7.66(5.04-11.66) ^*^ | 7.62(126.37) ^*^ | 7.61(5.35) ^*^ | 2.93(2.32) ^*^ |
| Feeling abnormal^*^ | 22 | 1.76(1.16-2.68) ^*^ | 1.76(7.21) | 1.76(1.24) | 0.81(0.21) ^*^ |
| Muscle spasms^*^ | 20 | 1.85(1.19-2.87) ^*^ | 1.84(7.75) | 1.84(1.28) | 0.88(0.25) ^*^ |
| Pain in extremity | 19 | 0.97(0.62-1.52) | 0.97(0.02) | 0.97(0.66) | -0.05(-0.7) |
| Chest pain^*^ | 18 | 1.63(1.03-2.59) ^*^ | 1.63(4.39) | 1.63(1.11) | 0.7(0.04) ^*^ |
| Pain | 18 | 0.68(0.43-1.08) | 0.68(2.74) | 0.68(0.46) | -0.56(-1.22) |
| Heart rate increased^*^ | 17 | 3.12(1.94-5.03) ^*^ | 3.11(24.37) ^*^ | 3.11(2.09) ^*^ | 1.64(0.95) ^*^ |
| Atrial fibrillation | 16 | 1.29(0.79-2.11) | 1.29(1.04) | 1.29(0.85) | 0.37(-0.34) |
| Pyrexia | 16 | 0.79(0.48-1.29) | 0.79(0.88) | 0.79(0.53) | -0.34(-1.04) |
| Tachycardia^*^ | 16 | 3.55(2.17-5.81) ^*^ | 3.54(29.2) ^*^ | 3.54(2.35) ^*^ | 1.82(1.12) ^*^ |
| Nervousness^*^ | 16 | 4.98(3.05-8.14) ^*^ | 4.96(50.61) ^*^ | 4.96(3.29) ^*^ | 2.31(1.61) ^*^ |
| Palpitations^*^ | 13 | 2.22(1.29-3.82) ^*^ | 2.21(8.64) ^*^ | 2.21(1.4) | 1.14(0.37) ^*^ |
| Abdominal discomfort | 13 | 1.43(0.83-2.46) | 1.43(1.66) | 1.43(0.9) | 0.51(-0.26) |
| Dehydration | 13 | 1.06(0.62-1.83) | 1.06(0.05) | 1.06(0.67) | 0.08(-0.69) |
| Respiratory failure^*^ | 13 | 1.97(1.14-3.39) ^*^ | 1.96(6.15) | 1.96(1.24) | 0.97(0.2) ^*^ |
| Arthralgia | 13 | 0.58(0.34-1.01) | 0.59(3.82) | 0.59(0.37) | -0.77(-1.54) |
| Myalgia | 12 | 1.11(0.63-1.95) | 1.11(0.12) | 1.11(0.69) | 0.15(-0.66) |
| Sleep disorder^*^ | 12 | 3.81(2.16-6.72) ^*^ | 3.8(24.74) ^*^ | 3.8(2.36) ^*^ | 1.92(1.12) ^*^ |
| Pruritus | 12 | 0.62(0.35-1.09) | 0.62(2.83) | 0.62(0.39) | -0.69(-1.49) |
| Chills | 11 | 1.54(0.85-2.77) | 1.53(2.04) | 1.53(0.93) | 0.62(-0.22) |
| Dyspepsia | 10 | 1.7(0.91-3.16) | 1.7(2.86) | 1.7(1.01) | 0.76(-0.11) |
| Hypotension | 10 | 0.57(0.31-1.07) | 0.57(3.17) | 0.57(0.34) | -0.8(-1.67) |
| Wheezing^*^ | 10 | 2.64(1.42-4.92) ^*^ | 2.64(10.19) ^*^ | 2.64(1.57) | 1.4(0.53) ^*^ |
| Panic attack^*^ | 10 | 11.21(6.02-20.88) ^*^ | 11.18(92.44) ^*^ | 11.15(6.63) ^*^ | 3.48(2.61) ^*^ |
| Influenza | 10 | 1.8(0.97-3.35) | 1.8(3.53) | 1.8(1.07) | 0.84(-0.03) |
| Product use issue | 10 | 1.04(0.56-1.94) | 1.04(0.02) | 1.04(0.62) | 0.06(-0.81) |
| Constipation | 10 | 0.63(0.34-1.17) | 0.63(2.18) | 0.63(0.38) | -0.67(-1.54) |
| Cardiac arrest | 9 | 1.47(0.76-2.82) | 1.47(1.33) | 1.47(0.85) | 0.55(-0.36) |

Asterisks (^*^) indicate statistically significant signals in algorithm. ROR, Reporting Odds Ratio; PRR, Proportional Reporting Ratio; EBGM, Empirical Bayesian Geometric Mean; EBGM05, the lower limit of the 95% CI of EBGM; IC, information component; IC025, the lower limit of the 95% CI of the IC; CI, confidence interval; PT, Preferred Term.

**Supplementary Table S8:** Top 50 most frequent adverse events at the PT level for roflumilast in patients aged 18-64 from FAERS data.

| PT | Case numbers | ROR (95%Cl) | PRR (χ^2^) | EBGM (EBGM05) | IC(IC025) |
| --- | --- | --- | --- | --- | --- |
| Diarrhea^*^ | 65 | 4.55(3.55-5.84) ^*^ | 4.4(172.16) ^*^ | 4.39(3.57) ^*^ | 2.14(1.77) ^*^ |
| Weight decreased^*^ | 52 | 8.3(6.29-10.95) ^*^ | 8.05(322.03) ^*^ | 8.04(6.38) ^*^ | 3.01(2.6) ^*^ |
| Dyspnea^*^ | 51 | 3.8(2.87-5.03) ^*^ | 3.7(101.61) ^*^ | 3.7(2.93) ^*^ | 1.89(1.48) ^*^ |
| Nausea^*^ | 50 | 2.28(1.72-3.02) ^*^ | 2.24(34.65) ^*^ | 2.24(1.77) | 1.16(0.75) ^*^ |
| Headache^*^ | 50 | 2.69(2.03-3.57) ^*^ | 2.63(51.35) ^*^ | 2.63(2.08) ^*^ | 1.4(0.99) ^*^ |
| Insomnia^*^ | 47 | 6.49(4.85-8.68) ^*^ | 6.32(211.29) ^*^ | 6.31(4.95) ^*^ | 2.66(2.24) ^*^ |
| Decreased appetite^*^ | 32 | 6.58(4.63-9.34) ^*^ | 6.46(148.05) ^*^ | 6.46(4.82) ^*^ | 2.69(2.18) ^*^ |
| Dizziness^*^ | 26 | 2.06(1.4-3.04) ^*^ | 2.04(13.93) ^*^ | 2.04(1.48) | 1.03(0.47) ^*^ |
| Anxiety^*^ | 26 | 3.09(2.09-4.55) ^*^ | 3.05(36.01) ^*^ | 3.05(2.2) ^*^ | 1.61(1.05) ^*^ |
| Suicidal ideation^*^ | 25 | 8.75(5.89-12.99) ^*^ | 8.62(168.59) ^*^ | 8.61(6.19) ^*^ | 3.11(2.54) ^*^ |
| Back pain^*^ | 25 | 4.08(2.75-6.07) ^*^ | 4.03(57.25) ^*^ | 4.03(2.9) ^*^ | 2.01(1.44) ^*^ |
| Tremor^*^ | 21 | 4.87(3.17-7.5) ^*^ | 4.82(63.7) ^*^ | 4.82(3.36) ^*^ | 2.27(1.65) ^*^ |
| Depression^*^ | 19 | 2.68(1.7-4.21) ^*^ | 2.65(19.69) ^*^ | 2.65(1.82) | 1.41(0.76) ^*^ |
| Asthenia^*^ | 19 | 2.17(1.38-3.42) ^*^ | 2.16(11.88) ^*^ | 2.16(1.48) | 1.11(0.46) ^*^ |
| Condition aggravated^*^ | 17 | 2.21(1.37-3.56) ^*^ | 2.19(11.09) | 2.19(1.47) | 1.13(0.45) ^*^ |
| Influenza like illness^*^ | 15 | 5.46(3.28-9.08) ^*^ | 5.42(54.09) ^*^ | 5.41(3.54) ^*^ | 2.44(1.71) ^*^ |
| Muscle spasms^*^ | 13 | 2.69(1.56-4.65) ^*^ | 2.68(13.72) ^*^ | 2.68(1.7) | 1.42(0.65) ^*^ |
| Vomiting | 12 | 0.93(0.53-1.64) | 0.93(0.06) | 0.93(0.58) | -0.1(-0.91) |
| Abdominal discomfort^*^ | 12 | 3.05(1.73-5.39) ^*^ | 3.04(16.43) ^*^ | 3.04(1.89) | 1.6(0.8) ^*^ |
| Chest pain^*^ | 11 | 1.93(1.07-3.49) ^*^ | 1.92(4.9) | 1.92(1.17) | 0.94(0.11) ^*^ |
| Malaise | 11 | 1.05(0.58-1.89) | 1.05(0.02) | 1.05(0.64) | 0.06(-0.77) |
| Fatigue | 11 | 0.54(0.3-0.99) | 0.55(4.16) | 0.55(0.33) | -0.87(-1.7) |
| Abdominal pain upper | 10 | 1.83(0.98-3.4) | 1.82(3.71) | 1.82(1.08) | 0.86(-0.01) |
| Myalgia^*^ | 10 | 2.18(1.17-4.06) ^*^ | 2.17(6.36) ^*^ | 2.17(1.29) | 1.12(0.25) ^*^ |
| Cough | 9 | 1.37(0.71-2.64) | 1.37(0.89) | 1.37(0.79) | 0.45(-0.46) |
| Pain | 9 | 0.58(0.3-1.12) | 0.58(2.73) | 0.58(0.34) | -0.78(-1.7) |
| Heart rate increased^*^ | 9 | 3.18(1.65-6.13) ^*^ | 3.17(13.38) ^*^ | 3.17(1.83) | 1.66(0.75) ^*^ |
| Nervousness^*^ | 9 | 6.55(3.4-12.62) ^*^ | 6.52(42.05) ^*^ | 6.51(3.76) ^*^ | 2.7(1.79) ^*^ |
| Chest discomfort^*^ | 8 | 2.55(1.27-5.1) ^*^ | 2.54(7.47) ^*^ | 2.54(1.42) | 1.34(0.38) ^*^ |
| Feeling abnormal | 8 | 1.27(0.64-2.55) | 1.27(0.46) | 1.27(0.71) | 0.35(-0.62) |
| Arthralgia | 8 | 0.7(0.35-1.4) | 0.7(1.03) | 0.7(0.39) | -0.51(-1.48) |
| Tachycardia^*^ | 7 | 2.45(1.17-5.15) ^*^ | 2.45(5.99) ^*^ | 2.45(1.31) | 1.29(0.27) ^*^ |
| Dyspepsia^*^ | 7 | 2.71(1.29-5.7) ^*^ | 2.7(7.53) ^*^ | 2.7(1.45) | 1.44(0.41) ^*^ |
| Drug ineffective | 7 | 0.27(0.13-0.57) | 0.27(13.77) | 0.27(0.15) | -1.87(-2.89) |
| Suicide attempt^*^ | 7 | 3.25(1.55-6.84) ^*^ | 3.24(10.87) ^*^ | 3.24(1.74) | 1.7(0.67) ^*^ |
| Palpitations | 7 | 1.95(0.93-4.11) | 1.95(3.25) | 1.95(1.05) | 0.96(-0.06) |
| Panic attack^*^ | 6 | 4.73(2.12-10.55) ^*^ | 4.72(17.59) ^*^ | 4.72(2.41) ^*^ | 2.24(1.14) ^*^ |
| Rash | 6 | 0.59(0.26-1.31) | 0.59(1.71) | 0.59(0.3) | -0.76(-1.85) |
| Nasopharyngitis | 6 | 1.17(0.53-2.61) | 1.17(0.15) | 1.17(0.6) | 0.23(-0.87) |
| Pain in extremity | 6 | 0.74(0.33-1.65) | 0.74(0.55) | 0.74(0.38) | -0.43(-1.53) |
| Respiratory failure^*^ | 6 | 3.49(1.56-7.78) ^*^ | 3.48(10.61) ^*^ | 3.48(1.78) | 1.8(0.71) ^*^ |
| Sleep disorder^*^ | 6 | 3.33(1.49-7.43) ^*^ | 3.32(9.74) ^*^ | 3.32(1.7) | 1.73(0.64) ^*^ |
| Abdominal pain | 6 | 0.84(0.38-1.88) | 0.85(0.17) | 0.85(0.43) | -0.24(-1.34) |
| Wheezing^*^ | 6 | 4.27(1.92-9.52) ^*^ | 4.26(14.96) ^*^ | 4.26(2.18) ^*^ | 2.09(1) ^*^ |
| Atrial fibrillation^*^ | 5 | 3.79(1.57-9.11) ^*^ | 3.78(10.21) ^*^ | 3.78(1.81) | 1.92(0.74) ^*^ |
| Pyrexia | 5 | 0.5(0.21-1.19) | 0.5(2.55) | 0.5(0.24) | -1.01(-2.19) |
| Product use issue | 5 | 1.49(0.62-3.58) | 1.49(0.8) | 1.49(0.71) | 0.57(-0.61) |
| Depressed mood^*^ | 5 | 3.22(1.34-7.75) ^*^ | 3.21(7.62) ^*^ | 3.21(1.54) | 1.68(0.5) ^*^ |
| Pneumothorax^*^ | 5 | 13.17(5.47-31.7) ^*^ | 13.13(55.99) ^*^ | 13.12(6.29) ^*^ | 3.71(2.53) ^*^ |
| Arrhythmia^*^ | 5 | 4.89(2.03-11.76) ^*^ | 4.87(15.39) ^*^ | 4.87(2.34) ^*^ | 2.28(1.1) ^*^ |

Asterisks (^*^) indicate statistically significant signals in algorithm. ROR, Reporting Odds Ratio; PRR, Proportional Reporting Ratio; EBGM, Empirical Bayesian Geometric Mean; EBGM05, the lower limit of the 95% CI of EBGM; IC, information component; IC025, the lower limit of the 95% CI of the IC; CI, confidence interval; PT, Preferred Term.

**Supplementary Table S9:** The adverse events at the PT level for roflumilast in patients aged under 18 from FAERS data.

| PT | Case numbers | ROR (95% Cl) | PRR (χ^2^) | EBGM (EBGM05) | IC(IC025) |
| --- | --- | --- | --- | --- | --- |
| Dyspnea | 1 | 14.42(1.89-110.24) | 13.46(11.6) | 13.46(2.45) | 3.75(1.58) |
| Oxygen saturation decreased | 1 | 47.91(6.27-366.35) | 44.56(42.64) | 44.54(8.12) | 5.48(3.3) |
| Headache | 1 | 8.9(1.16-68.05) | 8.34(6.51) | 8.34(1.52) | 3.06(0.89) |
| Respiratory distress | 1 | 48.12(6.29-368) | 44.76(42.84) | 44.74(8.16) | 5.48(3.31) |
| Ear infection | 1 | 73.96(9.67-565.64) | 68.75(66.8) | 68.71(12.52) | 6.1(3.93) |
| Sensitive skin | 1 | 261.3(34.13-2000.81) | 242.71(240.37) | 242.29(44.12) | 7.92(5.75) |
| Gastritis | 1 | 197.94(25.86-1515.02) | 183.87(181.71) | 183.63(33.45) | 7.52(5.35) |
| Decreased appetite | 1 | 22.31(2.92-170.61) | 20.79(18.9) | 20.79(3.79) | 4.38(2.21) |
| Sleep disorder | 1 | 55.16(7.21-421.8) | 51.29(49.36) | 51.27(9.35) | 5.68(3.51) |
| Dizziness | 1 | 19.88(2.6-151.99) | 18.53(16.65) | 18.53(3.38) | 4.21(2.04) |
| Nausea | 1 | 10.3(1.35-78.72) | 9.63(7.79) | 9.63(1.76) | 3.27(1.1) |

ROR, Reporting Odds Ratio; PRR, Proportional Reporting Ratio; EBGM, Empirical Bayesian Geometric Mean; EBGM05, the lower limit of the 95% CI of EBGM; IC, information component; IC025, the lower limit of the 95% CI of the IC; CI, confidence interval; PT, Preferred Term.

**Supplementary Table S10:** Top 50 most frequent adverse events at the PT level for oral roflumilast treatment from FAERS data.

| PT | Case numbers | ROR (95%Cl) | PRR (χ^2^) | EBGM (EBGM05) | IC (IC025) |
| --- | --- | --- | --- | --- | --- |
| Diarrhea^*^ | 384 | 4.75(4.29-5.26) ^*^ | 4.56(1079.77) ^*^ | 4.56(4.19) ^*^ | 2.19(2.04) ^*^ |
| Weight decreased^*^ | 305 | 8.99(8.02-10.08) ^*^ | 8.68(2079.1) ^*^ | 8.67(7.88) ^*^ | 3.12(2.95) ^*^ |
| Dyspnea^*^ | 278 | 3.94(3.5-4.44) ^*^ | 3.84(587.92) ^*^ | 3.83(3.47) ^*^ | 1.94(1.76) ^*^ |
| Nausea^*^ | 278 | 2.89(2.57-3.26) ^*^ | 2.83(331.91) ^*^ | 2.82(2.56) ^*^ | 1.5(1.32) ^*^ |
| Insomnia^*^ | 230 | 7.04(6.18-8.03) ^*^ | 6.87(1156.25) ^*^ | 6.86(6.15) ^*^ | 2.78(2.59) ^*^ |
| Decreased appetite^*^ | 204 | 6.84(5.95-7.86) ^*^ | 6.69(989.53) ^*^ | 6.68(5.95) ^*^ | 2.74(2.54) ^*^ |
| Headache^*^ | 183 | 2.28(1.97-2.65) ^*^ | 2.25(129.02) ^*^ | 2.25(1.99) | 1.17(0.96) ^*^ |
| Dizziness^*^ | 163 | 2.66(2.28-3.11) ^*^ | 2.62(165.15) ^*^ | 2.62(2.3) ^*^ | 1.39(1.16) ^*^ |
| Tremor^*^ | 115 | 5.72(4.75-6.87) ^*^ | 5.65(440.36) ^*^ | 5.64(4.84) ^*^ | 2.5(2.23) ^*^ |
| Back pain^*^ | 112 | 3.73(3.09-4.49) ^*^ | 3.69(220.31) ^*^ | 3.69(3.15) ^*^ | 1.88(1.61) ^*^ |
| Malaise^*^ | 108 | 1.82(1.5-2.2) ^*^ | 1.81(39.31) | 1.81(1.54) | 0.85(0.58) ^*^ |
| Anxiety^*^ | 107 | 2.92(2.42-3.54) ^*^ | 2.9(133.55) ^*^ | 2.9(2.47) ^*^ | 1.53(1.25) ^*^ |
| Suicidal ideation^*^ | 97 | 9.19(7.52-11.23) ^*^ | 9.09(697.89) ^*^ | 9.07(7.67) ^*^ | 3.18(2.89) ^*^ |
| Asthenia^*^ | 85 | 1.79(1.45-2.22) ^*^ | 1.78(29.32) | 1.78(1.49) | 0.83(0.52) ^*^ |
| Depression^*^ | 83 | 2.98(2.4-3.7) ^*^ | 2.96(107.88) ^*^ | 2.96(2.47) ^*^ | 1.56(1.25) ^*^ |
| Fatigue | 74 | 0.71(0.57-0.89) | 0.71(8.61) | 0.71(0.59) | -0.49(-0.82) |
| Condition aggravated^*^ | 67 | 1.77(1.39-2.26) ^*^ | 1.77(22.4) | 1.77(1.44) | 0.82(0.47) ^*^ |
| Cough^*^ | 66 | 1.8(1.41-2.29) ^*^ | 1.79(23.17) | 1.79(1.46) | 0.84(0.49) ^*^ |
| Feeling abnormal^*^ | 66 | 2.11(1.65-2.68) ^*^ | 2.1(37.97) ^*^ | 2.1(1.71) | 1.07(0.71) ^*^ |
| Vomiting | 64 | 1.11(0.87-1.42) | 1.11(0.71) | 1.11(0.9) | 0.15(-0.21) |
| Abdominal pain upper^*^ | 55 | 2.11(1.62-2.75) ^*^ | 2.1(31.7) ^*^ | 2.1(1.68) | 1.07(0.68) ^*^ |
| Influenza like illness^*^ | 53 | 5.05(3.85-6.62) ^*^ | 5.02(170.76) ^*^ | 5.02(4) ^*^ | 2.33(1.93) ^*^ |
| Abdominal discomfort^*^ | 45 | 1.98(1.48-2.65) ^*^ | 1.97(21.61) | 1.97(1.54) | 0.98(0.55) ^*^ |
| Muscle spasms^*^ | 44 | 1.84(1.37-2.48) ^*^ | 1.84(16.9) | 1.84(1.44) | 0.88(0.45) ^*^ |
| Drug ineffective | 43 | 0.24(0.18-0.32) | 0.24(104.66) | 0.24(0.19) | -2.05(-2.48) |
| Heart rate increased^*^ | 42 | 3.54(2.62-4.8) ^*^ | 3.53(76.21) ^*^ | 3.53(2.74) ^*^ | 1.82(1.38) ^*^ |
| Abdominal pain^*^ | 40 | 1.37(1.01-1.88) ^*^ | 1.37(4.06) | 1.37(1.06) | 0.46(0.01) ^*^ |
| Pain | 38 | 0.45(0.32-0.61) | 0.45(26) | 0.45(0.34) | -1.16(-1.62) |
| Chest pain^*^ | 37 | 1.68(1.22-2.32) ^*^ | 1.68(10.15) | 1.68(1.28) | 0.75(0.28) ^*^ |
| Nervousness^*^ | 37 | 5.79(4.19-7.99) ^*^ | 5.76(145.63) ^*^ | 5.76(4.39) ^*^ | 2.53(2.06) ^*^ |
| Pain in extremity | 35 | 0.89(0.64-1.24) | 0.89(0.46) | 0.89(0.68) | -0.17(-0.65) |
| Myalgia^*^ | 34 | 1.64(1.17-2.29) ^*^ | 1.63(8.35) | 1.63(1.23) | 0.71(0.22) ^*^ |
| Atrial fibrillation^*^ | 32 | 2.62(1.85-3.71) ^*^ | 2.62(31.96) ^*^ | 2.61(1.96) | 1.39(0.88) ^*^ |
| Palpitations^*^ | 31 | 2.16(1.52-3.07) ^*^ | 2.15(19.17) ^*^ | 2.15(1.6) | 1.11(0.59) ^*^ |
| Product use issue | 31 | 1.17(0.82-1.66) | 1.17(0.73) | 1.17(0.87) | 0.22(-0.29) |
| Sleep disorder^*^ | 29 | 3.28(2.28-4.73) ^*^ | 3.27(45.82) ^*^ | 3.27(2.41) ^*^ | 1.71(1.18) ^*^ |
| Rash | 27 | 0.48(0.33-0.71) | 0.49(14.74) | 0.49(0.35) | -1.04(-1.59) |
| Tachycardia^*^ | 27 | 2.54(1.74-3.7) ^*^ | 2.53(25.06) ^*^ | 2.53(1.85) | 1.34(0.79) ^*^ |
| Gastrointestinal disorder^*^ | 25 | 2.31(1.56-3.42) ^*^ | 2.3(18.44) ^*^ | 2.3(1.66) | 1.2(0.64) ^*^ |
| Pruritus | 24 | 0.51(0.34-0.76) | 0.51(11.11) | 0.51(0.37) | -0.96(-1.54) |
| Product dose omission issue | 24 | 0.71(0.47-1.06) | 0.71(2.89) | 0.71(0.51) | -0.5(-1.07) |
| Dehydration | 23 | 1.42(0.94-2.14) | 1.42(2.88) | 1.42(1.01) | 0.51(-0.08) |
| Pyrexia | 23 | 0.53(0.35-0.79) | 0.53(9.81) | 0.53(0.37) | -0.92(-1.51) |
| Respiratory failure^*^ | 23 | 2.63(1.75-3.96) ^*^ | 2.62(23.12) ^*^ | 2.62(1.86) | 1.39(0.8) ^*^ |
| Arthralgia | 23 | 0.42(0.28-0.63) | 0.42(18.5) | 0.42(0.3) | -1.25(-1.84) |
| Panic attack^*^ | 22 | 4.98(3.28-7.57) ^*^ | 4.97(69.69) ^*^ | 4.96(3.5) ^*^ | 2.31(1.71) ^*^ |
| Lung disorder^*^ | 21 | 3.37(2.19-5.17) ^*^ | 3.36(34.86) ^*^ | 3.36(2.35) ^*^ | 1.75(1.13) ^*^ |
| Chest discomfort^*^ | 21 | 1.65(1.08-2.53) ^*^ | 1.65(5.38) | 1.65(1.15) | 0.72(0.11) ^*^ |
| Productive cough^*^ | 20 | 3.22(2.08-4.99) ^*^ | 3.21(30.49) ^*^ | 3.21(2.22) ^*^ | 1.68(1.05) ^*^ |
| Hallucination^*^ | 20 | 2.22(1.43-3.45) ^*^ | 2.22(13.4) ^*^ | 2.22(1.54) | 1.15(0.52) ^*^ |

Asterisks (^*^) indicate statistically significant signals in algorithm. ROR, Reporting Odds Ratio; PRR, Proportional Reporting Ratio; EBGM, Empirical Bayesian Geometric Mean; EBGM05, the lower limit of the 95% CI of EBGM; IC, information component; IC025, the lower limit of the 95% CI of the IC; CI, confidence interval; PT, Preferred Term.

**Supplementary Table S11:** Top 50 most frequent adverse events at the PT level for topical roflumilast treatment from FAERS data.

| PT | Case numbers | ROR (95%Cl) | PRR (χ^2^) | EBGM (EBGM05) | IC (IC025) |
| --- | --- | --- | --- | --- | --- |
| Headache^*^ | 30 | 6.32(4.37-9.15) ^*^ | 5.99(126.07) ^*^ | 5.99(4.4) ^*^ | 2.58(2.05) ^*^ |
| Diarrhoea^*^ | 29 | 5.88(4.04-8.56) ^*^ | 5.59(110.4) ^*^ | 5.59(4.08) ^*^ | 2.48(1.94) ^*^ |
| Drug ineffective^*^ | 21 | 1.96(1.26-3.03) ^*^ | 1.91(9.39) | 1.91(1.33) | 0.94(0.31) ^*^ |
| Insomnia^*^ | 15 | 7.46(4.46-12.47) ^*^ | 7.25(81.23) ^*^ | 7.25(4.72) ^*^ | 2.86(2.13) ^*^ |
| Skin burning sensation^*^ | 15 | 25.14(15.03-42.04) ^*^ | 24.39(336.8) ^*^ | 24.38(15.86) ^*^ | 4.61(3.87) ^*^ |
| Pruritus^*^ | 12 | 4.25(2.39-7.53) ^*^ | 4.17(29.05) ^*^ | 4.17(2.58) ^*^ | 2.06(1.25) ^*^ |
| Nausea^*^ | 11 | 1.83(1.01-3.33) ^*^ | 1.81(4.06) | 1.81(1.1) | 0.86(0.01) ^*^ |
| Skin exfoliation^*^ | 10 | 14.85(7.94-27.78) ^*^ | 14.56(126.48) ^*^ | 14.56(8.62) ^*^ | 3.86(2.98) ^*^ |
| Inappropriate schedule of product administration^*^ | 9 | 6.46(3.34-12.49) ^*^ | 6.36(40.76) ^*^ | 6.36(3.66) ^*^ | 2.67(1.75) ^*^ |
| Application site pain^*^ | 8 | 41.68(20.72-83.85) ^*^ | 41.01(312.24) ^*^ | 40.99(22.84) ^*^ | 5.36(4.39) ^*^ |
| Pain of skin^*^ | 8 | 34.17(16.99-68.74) ^*^ | 33.62(253.24) ^*^ | 33.61(18.73) ^*^ | 5.07(4.1) ^*^ |
| Condition aggravated^*^ | 8 | 3.46(1.72-6.96) ^*^ | 3.42(13.77) ^*^ | 3.42(1.91) | 1.77(0.8) ^*^ |
| Dermatitis contact^*^ | 7 | 79.59(37.73-167.91) ^*^ | 78.45(534.89) ^*^ | 78.39(41.97) ^*^ | 6.29(5.26) ^*^ |
| Therapeutic product effect incomplete^*^ | 7 | 11.52(5.46-24.31) ^*^ | 11.37(66.29) ^*^ | 11.37(6.09) ^*^ | 3.51(2.48) ^*^ |
| Vision blurred^*^ | 5 | 4.9(2.03-11.84) ^*^ | 4.86(15.38) ^*^ | 4.86(2.33) ^*^ | 2.28(1.1) ^*^ |
| Urinary tract infection^*^ | 5 | 3.64(1.51-8.78) ^*^ | 3.61(9.46) ^*^ | 3.61(1.73) | 1.85(0.67) ^*^ |
| Application site erythema^*^ | 5 | 23.43(9.71-56.56) ^*^ | 23.2(106.23) ^*^ | 23.19(11.09) ^*^ | 4.54(3.35) ^*^ |
| Weight decreased | 4 | 1.85(0.69-4.95) | 1.84(1.55) | 1.84(0.81) | 0.88(-0.41) |
| Rash | 4 | 1.17(0.44-3.13) | 1.17(0.1) | 1.17(0.51) | 0.22(-1.07) |
| Skin discolouration^*^ | 4 | 10.89(4.07-29.15) ^*^ | 10.81(35.64) ^*^ | 10.81(4.74) ^*^ | 3.43(2.14) ^*^ |
| Application site pruritus^*^ | 4 | 21.88(8.18-58.54) ^*^ | 21.7(79.01) ^*^ | 21.7(9.52) ^*^ | 4.44(3.14) ^*^ |
| Heart rate increased^*^ | 4 | 5.49(2.05-14.68) ^*^ | 5.45(14.55) ^*^ | 5.45(2.39) ^*^ | 2.45(1.15) ^*^ |
| Skin irritation^*^ | 4 | 9.77(3.65-26.13) ^*^ | 9.69(31.21) ^*^ | 9.69(4.25) ^*^ | 3.28(1.98) ^*^ |
| Urticaria^*^ | 4 | 3.18(1.19-8.52) ^*^ | 3.16(5.94) ^*^ | 3.16(1.39) | 1.66(0.36) ^*^ |
| Erythema | 4 | 2.41(0.9-6.44) | 2.4(3.27) | 2.4(1.05) | 1.26(-0.04) |
| Lip swelling^*^ | 3 | 11.55(3.71-35.96) ^*^ | 11.49(28.74) ^*^ | 11.49(4.44) ^*^ | 3.52(2.07) ^*^ |
| Dizziness | 3 | 0.78(0.25-2.43) | 0.78(0.18) | 0.78(0.3) | -0.35(-1.8) |
| Folliculitis^*^ | 3 | 38.94(12.51-121.19) ^*^ | 38.7(110.15) ^*^ | 38.69(14.96) ^*^ | 5.27(3.82) ^*^ |
| Skin hyperpigmentation^*^ | 3 | 43.77(14.06-136.23) ^*^ | 43.5(124.54) ^*^ | 43.48(16.82) ^*^ | 5.44(3.99) ^*^ |
| Product use issue | 3 | 1.83(0.59-5.71) | 1.83(1.13) | 1.83(0.71) | 0.87(-0.58) |
| Pain | 3 | 0.57(0.18-1.78) | 0.57(0.96) | 0.57(0.22) | -0.8(-2.25) |
| Paraesthesia oral^*^ | 3 | 27.33(8.78-85.06) ^*^ | 27.17(75.61) ^*^ | 27.16(10.51) ^*^ | 4.76(3.31) ^*^ |
| Palpitations^*^ | 3 | 3.39(1.09-10.56) ^*^ | 3.38(5.03) ^*^ | 3.38(1.31) | 1.76(0.31) ^*^ |
| Dry skin | 3 | 2.73(0.88-8.49) | 2.72(3.27) | 2.72(1.05) | 1.44(-0.01) |
| Dermatitis^*^ | 3 | 19.01(6.11-59.16) ^*^ | 18.9(50.86) ^*^ | 18.89(7.31) ^*^ | 4.24(2.79) ^*^ |
| Blood pressure increased | 3 | 2.52(0.81-7.85) | 2.51(2.74) | 2.51(0.97) | 1.33(-0.12) |
| Rash erythematous^*^ | 3 | 8.98(2.88-27.93) ^*^ | 8.93(21.13) ^*^ | 8.93(3.45) ^*^ | 3.16(1.71) ^*^ |
| Anaphylactic reaction | 2 | 4.96(1.24-19.88) | 4.94(6.29) | 4.94(1.55) | 2.3(0.63) |
| Asthenia | 2 | 0.68(0.17-2.72) | 0.68(0.3) | 0.68(0.21) | -0.56(-2.23) |
| Arthralgia | 2 | 0.59(0.15-2.37) | 0.59(0.56) | 0.59(0.19) | -0.75(-2.43) |
| Paraesthesia | 2 | 1.63(0.41-6.53) | 1.63(0.48) | 1.63(0.51) | 0.7(-0.97) |
| Application site irritation | 2 | 20.93(5.22-83.94) | 20.85(37.79) | 20.84(6.52) ^*^ | 4.38(2.71) |
| Skin fissures | 2 | 13.69(3.41-54.9) | 13.64(23.42) | 13.63(4.26) ^*^ | 3.77(2.1) |
| Hypersensitivity | 2 | 1.35(0.34-5.4) | 1.35(0.18) | 1.35(0.42) | 0.43(-1.24) |
| Pyrexia | 2 | 0.74(0.19-2.98) | 0.74(0.18) | 0.74(0.23) | -0.43(-2.1) |
| Hypoaesthesia oral | 2 | 17.61(4.39-70.64) | 17.54(31.2) | 17.54(5.49) ^*^ | 4.13(2.46) |
| Cyst | 2 | 19.88(4.96-79.72) | 19.8(35.7) | 19.79(6.19) ^*^ | 4.31(2.64) |
| Swelling | 2 | 2.62(0.65-10.51) | 2.61(2) | 2.61(0.82) | 1.39(-0.28) |
| Influenza like illness | 2 | 3.08(0.77-12.35) | 3.07(2.8) | 3.07(0.96) | 1.62(-0.05) |
| Acne | 2 | 2.93(0.73-11.76) | 2.93(2.54) | 2.93(0.92) | 1.55(-0.12) |

Asterisks (^*^) indicate statistically significant signals in algorithm. ROR, Reporting Odds Ratio; PRR, Proportional Reporting Ratio; EBGM, Empirical Bayesian Geometric Mean; EBGM05, the lower limit of the 95% CI of EBGM; IC, information component; IC025, the lower limit of the 95% CI of the IC; CI, confidence interval; PT, Preferred Term.

**Supplementary Table S12:** Top 100 most frequent adverse events at the PT level for roflumilast excluding common medication co-usage from FAERS data.

| PT | Case numbers | ROR (95%Cl) | PRR (χ^2^) | EBGM (EBGM05) | IC (IC025) |
| --- | --- | --- | --- | --- | --- |
| Diarrhoea^*^ | 422 | 4.96(4.5-5.47) ^*^ | 4.76(1265.36) ^*^ | 4.76(4.38) ^*^ | 2.25(2.11) ^*^ |
| Weight decreased^*^ | 314 | 8.32(7.43-9.31) ^*^ | 8.04(1942.87) ^*^ | 8.03(7.31) ^*^ | 3.01(2.84) ^*^ |
| Nausea^*^ | 295 | 2.76(2.45-3.1) ^*^ | 2.69(318.29) ^*^ | 2.69(2.44) ^*^ | 1.43(1.26) ^*^ |
| Dyspnoea^*^ | 281 | 3.64(3.23-4.1) ^*^ | 3.55(519.83) ^*^ | 3.55(3.21) ^*^ | 1.83(1.65) ^*^ |
| Insomnia^*^ | 248 | 6.74(5.94-7.65) ^*^ | 6.57(1176.16) ^*^ | 6.57(5.91) ^*^ | 2.72(2.53) ^*^ |
| Headache^*^ | 217 | 2.51(2.19-2.87) ^*^ | 2.47(192.22) ^*^ | 2.47(2.21) ^*^ | 1.31(1.11) ^*^ |
| Decreased appetite^*^ | 208 | 6.69(5.83-7.67) ^*^ | 6.55(980.01) ^*^ | 6.54(5.83) ^*^ | 2.71(2.51) ^*^ |
| Dizziness^*^ | 166 | 2.41(2.07-2.81) ^*^ | 2.38(134.39) ^*^ | 2.38(2.1) ^*^ | 1.25(1.03) ^*^ |
| Tremor^*^ | 119 | 5.11(4.27-6.13) ^*^ | 5.06(387.92) ^*^ | 5.05(4.34) ^*^ | 2.34(2.07) ^*^ |
| Back pain^*^ | 114 | 3.52(2.92-4.23) ^*^ | 3.48(202.38) ^*^ | 3.48(2.98) ^*^ | 1.8(1.53) ^*^ |
| Anxiety^*^ | 109 | 2.72(2.25-3.28) ^*^ | 2.69(116.68) ^*^ | 2.69(2.3) ^*^ | 1.43(1.15) ^*^ |
| Malaise^*^ | 109 | 1.76(1.45-2.12) ^*^ | 1.75(35) | 1.75(1.49) | 0.8(0.53) ^*^ |
| Suicidal ideation^*^ | 99 | 7.76(6.37-9.46) ^*^ | 7.68(575.57) ^*^ | 7.67(6.5) ^*^ | 2.94(2.65) ^*^ |
| Asthenia^*^ | 87 | 1.66(1.34-2.05) ^*^ | 1.65(22.42) | 1.65(1.38) | 0.72(0.41) ^*^ |
| Depression^*^ | 83 | 2.55(2.05-3.17) ^*^ | 2.54(77.43) ^*^ | 2.53(2.12) ^*^ | 1.34(1.03) ^*^ |
| Fatigue | 76 | 0.7(0.56-0.88) | 0.7(9.58) | 0.7(0.58) | -0.51(-0.84) |
| Condition aggravated^*^ | 76 | 1.88(1.5-2.36) ^*^ | 1.87(31.16) | 1.87(1.55) | 0.91(0.58) ^*^ |
| Cough^*^ | 70 | 1.82(1.44-2.31) ^*^ | 1.82(25.83) | 1.82(1.49) | 0.86(0.52) ^*^ |
| Vomiting | 67 | 1.04(0.81-1.32) | 1.04(0.08) | 1.04(0.85) | 0.05(-0.3) |
| Feeling abnormal^*^ | 67 | 1.95(1.53-2.48) ^*^ | 1.94(30.72) | 1.94(1.59) | 0.96(0.61) ^*^ |
| Drug ineffective | 65 | 0.35(0.27-0.45) | 0.35(78.44) | 0.35(0.29) | -1.5(-1.86) |
| Abdominal pain upper^*^ | 56 | 1.98(1.52-2.58) ^*^ | 1.98(27.06) | 1.98(1.59) | 0.98(0.6) ^*^ |
| Influenza like illness^*^ | 55 | 4.68(3.59-6.11) ^*^ | 4.66(158.21) ^*^ | 4.66(3.73) ^*^ | 2.22(1.83) ^*^ |
| Abdominal discomfort^*^ | 46 | 2(1.49-2.67) ^*^ | 1.99(22.77) | 1.99(1.56) | 0.99(0.57) ^*^ |
| Muscle spasms^*^ | 46 | 1.78(1.33-2.38) ^*^ | 1.78(15.73) | 1.78(1.4) | 0.83(0.41) ^*^ |
| Heart rate increased^*^ | 46 | 3.34(2.5-4.47) ^*^ | 3.33(75.08) ^*^ | 3.33(2.61) ^*^ | 1.73(1.31) ^*^ |
| Abdominal pain | 40 | 1.24(0.91-1.69) | 1.24(1.82) | 1.24(0.95) | 0.31(-0.15) |
| Pain | 40 | 0.46(0.33-0.62) | 0.46(25.96) | 0.46(0.35) | -1.13(-1.58) |
| Chest pain^*^ | 39 | 1.48(1.08-2.03) ^*^ | 1.48(6.11) | 1.48(1.14) | 0.57(0.11) ^*^ |
| Nervousness^*^ | 37 | 4.89(3.54-6.75) ^*^ | 4.87(113.89) ^*^ | 4.87(3.72) ^*^ | 2.28(1.81) ^*^ |
| Pruritus | 37 | 0.75(0.54-1.03) | 0.75(3.19) | 0.75(0.57) | -0.42(-0.89) |
| Myalgia^*^ | 35 | 1.47(1.05-2.05) ^*^ | 1.47(5.23) | 1.47(1.11) | 0.55(0.07) ^*^ |
| Palpitations^*^ | 35 | 2.15(1.54-2.99) ^*^ | 2.14(21.36) ^*^ | 2.14(1.62) | 1.1(0.62) ^*^ |
| Pain in extremity | 34 | 0.81(0.57-1.13) | 0.81(1.6) | 0.81(0.61) | -0.31(-0.8) |
| Product use issue | 32 | 1.27(0.9-1.79) | 1.27(1.8) | 1.27(0.95) | 0.34(-0.16) |
| Rash | 31 | 0.52(0.37-0.74) | 0.52(13.49) | 0.52(0.39) | -0.93(-1.44) |
| Atrial fibrillation^*^ | 31 | 2.27(1.59-3.22) ^*^ | 2.26(21.82) ^*^ | 2.26(1.68) | 1.18(0.67) ^*^ |
| Sleep disorder^*^ | 29 | 3.05(2.12-4.4) ^*^ | 3.05(39.86) ^*^ | 3.04(2.24) ^*^ | 1.61(1.08) ^*^ |
| Tachycardia^*^ | 28 | 2.26(1.56-3.28) ^*^ | 2.26(19.61) ^*^ | 2.26(1.65) | 1.17(0.64) ^*^ |
| Pyrexia | 27 | 0.55(0.38-0.8) | 0.55(9.87) | 0.55(0.4) | -0.86(-1.4) |
| Gastrointestinal disorder^*^ | 27 | 2.35(1.61-3.44) ^*^ | 2.35(20.97) ^*^ | 2.35(1.71) | 1.23(0.69) ^*^ |
| Arthralgia | 26 | 0.45(0.31-0.66) | 0.45(17.23) | 0.45(0.33) | -1.14(-1.7) |
| Product dose omission issue | 25 | 0.78(0.52-1.15) | 0.78(1.6) | 0.78(0.56) | -0.36(-0.93) |
| Dehydration | 23 | 1.22(0.81-1.84) | 1.22(0.93) | 1.22(0.87) | 0.29(-0.3) |
| Respiratory failure^*^ | 23 | 2.24(1.49-3.37) ^*^ | 2.23(15.67) ^*^ | 2.23(1.59) | 1.16(0.57) ^*^ |
| Panic attack^*^ | 22 | 4.3(2.83-6.54) ^*^ | 4.29(55.56) ^*^ | 4.29(3.02) ^*^ | 2.1(1.5) ^*^ |
| Chest discomfort^*^ | 22 | 1.57(1.03-2.38) ^*^ | 1.56(4.47) | 1.56(1.1) | 0.64(0.04) ^*^ |
| Lung disorder^*^ | 21 | 3.11(2.03-4.78) ^*^ | 3.11(30.02) ^*^ | 3.11(2.17) ^*^ | 1.64(1.02) ^*^ |
| Productive cough^*^ | 21 | 3.3(2.15-5.06) ^*^ | 3.29(33.52) ^*^ | 3.29(2.3) ^*^ | 1.72(1.1) ^*^ |
| Dyspepsia | 20 | 1.48(0.96-2.3) | 1.48(3.15) | 1.48(1.03) | 0.57(-0.06) |
| Depressed mood^*^ | 20 | 2.77(1.79-4.3) ^*^ | 2.77(22.64) ^*^ | 2.77(1.92) | 1.47(0.84) ^*^ |
| Wheezing^*^ | 20 | 2.52(1.63-3.92) ^*^ | 2.52(18.36) ^*^ | 2.52(1.75) | 1.33(0.7) ^*^ |
| Vision blurred | 20 | 1.06(0.68-1.65) | 1.06(0.07) | 1.06(0.73) | 0.09(-0.55) |
| Memory impairment | 20 | 1.03(0.66-1.6) | 1.03(0.02) | 1.03(0.71) | 0.04(-0.59) |
| Myocardial infarction | 19 | 0.74(0.47-1.16) | 0.74(1.77) | 0.74(0.51) | -0.44(-1.08) |
| Nightmare^*^ | 19 | 3.86(2.46-6.05) ^*^ | 3.85(40.11) ^*^ | 3.85(2.64) ^*^ | 1.94(1.3) ^*^ |
| Hallucination^*^ | 19 | 1.84(1.17-2.89) ^*^ | 1.84(7.28) | 1.84(1.26) | 0.88(0.23) ^*^ |
| Suicide attempt^*^ | 19 | 2.23(1.42-3.5) ^*^ | 2.23(12.88) ^*^ | 2.23(1.53) | 1.16(0.51) ^*^ |
| Confusional state | 18 | 0.79(0.5-1.25) | 0.79(1.03) | 0.79(0.54) | -0.34(-1.01) |
| Restlessness^*^ | 17 | 3.28(2.04-5.28) ^*^ | 3.28(26.91) ^*^ | 3.28(2.2) ^*^ | 1.71(1.03) ^*^ |
| Hypotension | 17 | 0.6(0.38-0.97) | 0.6(4.41) | 0.6(0.41) | -0.73(-1.41) |
| Lung neoplasm malignant^*^ | 17 | 2.85(1.77-4.58) ^*^ | 2.84(20.29) ^*^ | 2.84(1.91) | 1.51(0.83) ^*^ |
| Oedema peripheral | 17 | 0.97(0.6-1.55) | 0.97(0.02) | 0.97(0.65) | -0.05(-0.73) |
| Drug intolerance | 17 | 1.29(0.8-2.07) | 1.29(1.09) | 1.29(0.86) | 0.36(-0.32) |
| Crying^*^ | 16 | 3.03(1.86-4.96) ^*^ | 3.03(21.77) ^*^ | 3.03(2.01) ^*^ | 1.6(0.9) ^*^ |
| Rhinorrhoea^*^ | 16 | 1.82(1.11-2.97) ^*^ | 1.81(5.86) | 1.81(1.2) | 0.86(0.16) ^*^ |
| Gastrooesophageal reflux disease | 16 | 1.45(0.89-2.37) | 1.45(2.23) | 1.45(0.96) | 0.54(-0.17) |
| Gait disturbance | 16 | 0.59(0.36-0.96) | 0.59(4.67) | 0.59(0.39) | -0.77(-1.47) |
| Chills | 16 | 0.96(0.59-1.58) | 0.96(0.02) | 0.96(0.64) | -0.05(-0.75) |
| Skin burning sensation | 16 | 1.61(0.99-2.63) | 1.61(3.71) | 1.61(1.07) | 0.69(-0.01) |
| Abdominal distension | 15 | 1.05(0.63-1.74) | 1.05(0.03) | 1.05(0.69) | 0.07(-0.65) |
| Visual impairment | 15 | 0.9(0.54-1.49) | 0.9(0.18) | 0.9(0.59) | -0.16(-0.88) |
| Muscular weakness | 15 | 0.94(0.57-1.56) | 0.94(0.06) | 0.94(0.61) | -0.09(-0.81) |
| Hyperhidrosis | 15 | 0.82(0.49-1.36) | 0.82(0.6) | 0.82(0.54) | -0.29(-1.01) |
| Drug interaction | 15 | 0.67(0.4-1.11) | 0.67(2.43) | 0.67(0.44) | -0.58(-1.3) |
| Influenza | 15 | 1.02(0.61-1.69) | 1.02(0) | 1.02(0.67) | 0.02(-0.7) |
| Fall | 15 | 0.32(0.19-0.53) | 0.32(21.55) | 0.32(0.21) | -1.64(-2.36) |
| Feeling jittery^*^ | 15 | 5.51(3.32-9.14) ^*^ | 5.5(55.16) ^*^ | 5.49(3.59) ^*^ | 2.46(1.74) ^*^ |
| Mood altered^*^ | 15 | 3.97(2.39-6.6) ^*^ | 3.97(33.3) ^*^ | 3.97(2.6) ^*^ | 1.99(1.27) ^*^ |
| Paraesthesia | 15 | 0.67(0.4-1.11) | 0.67(2.48) | 0.67(0.44) | -0.58(-1.3) |
| Nasopharyngitis | 14 | 0.55(0.33-0.93) | 0.55(5.12) | 0.55(0.36) | -0.86(-1.6) |
| Hypersensitivity | 14 | 0.54(0.32-0.91) | 0.54(5.47) | 0.54(0.35) | -0.89(-1.63) |
| Agitation | 14 | 1.33(0.79-2.25) | 1.33(1.16) | 1.33(0.86) | 0.41(-0.33) |
| Erythema | 14 | 0.49(0.29-0.82) | 0.49(7.52) | 0.49(0.32) | -1.03(-1.78) |
| Skin exfoliation | 14 | 1.24(0.74-2.1) | 1.24(0.67) | 1.24(0.8) | 0.31(-0.43) |
| Syncope | 13 | 0.92(0.53-1.58) | 0.92(0.09) | 0.92(0.58) | -0.12(-0.89) |
| Anaemia | 13 | 0.48(0.28-0.83) | 0.48(7.35) | 0.48(0.3) | -1.06(-1.83) |
| Urticaria | 13 | 0.57(0.33-0.99) | 0.57(4.11) | 0.57(0.36) | -0.8(-1.57) |
| Somnolence | 13 | 0.46(0.27-0.79) | 0.46(8.17) | 0.46(0.29) | -1.11(-1.89) |
| Cerebrovascular accident | 13 | 0.53(0.31-0.92) | 0.53(5.28) | 0.53(0.34) | -0.9(-1.67) |
| Cardiac disorder | 13 | 0.98(0.57-1.68) | 0.98(0.01) | 0.98(0.62) | -0.04(-0.81) |
| Constipation | 13 | 0.44(0.26-0.76) | 0.44(9.05) | 0.44(0.28) | -1.17(-1.94) |
| Infection | 13 | 0.66(0.38-1.14) | 0.66(2.28) | 0.66(0.42) | -0.6(-1.37) |
| Epistaxis | 12 | 1.14(0.65-2) | 1.14(0.2) | 1.14(0.71) | 0.18(-0.62) |
| Cardiac arrest | 12 | 1.03(0.58-1.81) | 1.02(0.01) | 1.02(0.64) | 0.04(-0.77) |
| Neoplasm malignant | 12 | 1.3(0.74-2.3) | 1.3(0.85) | 1.3(0.81) | 0.38(-0.42) |
| Pancreatitis | 12 | 1.62(0.92-2.86) | 1.62(2.87) | 1.62(1.01) | 0.7(-0.1) |
| Urinary tract infection | 12 | 0.51(0.29-0.89) | 0.51(5.73) | 0.51(0.32) | -0.98(-1.78) |
| Pollakiuria^*^ | 12 | 2.07(1.18-3.65) ^*^ | 2.07(6.67) ^*^ | 2.07(1.29) | 1.05(0.25) ^*^ |
| Abnormal dreams^*^ | 12 | 3(1.7-5.28) ^*^ | 2.99(15.93) ^*^ | 2.99(1.86) ^*^ | 1.58(0.78) ^*^ |

Asterisks (^*^) indicate statistically significant signals in algorithm. ROR, Reporting Odds Ratio; PRR, Proportional Reporting Ratio; EBGM, Empirical Bayesian Geometric Mean; EBGM05, the lower limit of the 95% CI of EBGM; IC, information component; IC025, the lower limit of the 95% CI of the IC; CI, confidence interval; PT, Preferred Term.
